# Unifying Candidate Gene and GWAS Approaches in Asthma

**DOI:** 10.1371/journal.pone.0013894

**Published:** 2010-11-12

**Authors:** Sven Michel, Liming Liang, Martin Depner, Norman Klopp, Andreas Ruether, Ashish Kumar, Michaela Schedel, Christian Vogelberg, Erika von Mutius, Andrea von Berg, Albrecht Bufe, Ernst Rietschel, Andrea Heinzmann, Otto Laub, Burkhard Simma, Thomas Frischer, Jon Genuneit, Ivo G. Gut, Stefan Schreiber, Mark Lathrop, Thomas Illig, Michael Kabesch

**Affiliations:** 1 Center for Pediatrics, Clinic for Pediatric Pneumology, Allergology and Neonatology, Hannover Medical School, Hannover, Germany; 2 Center for Statistical Genetics, Department of Biostatistics, University of Michigan, Ann Arbor, Michigan, United States of America; 3 University Children's Hospital, Ludwig Maximilian's University, Munich, Germany; 4 Institute of Epidemiology, Helmholtz Centre Munich, Neuherberg, Germany; 5 Institute of Clinical Molecular Biology, University Hospital Schleswig-Holstein, Campus Kiel, Kiel, Germany; 6 Wellcome Trust Centre for Human Genetics, University of Oxford, Oxford, United Kingdom; 7 University Children's Hospital, Technical University Dresden, Dresden, Germany; 8 Research Institute for the Prevention of Allergic Diseases, Children's Department, Marien-Hospital, Wesel, Germany; 9 Department of Experimental Pneumology, Ruhr-University, Bochum, Germany; 10 University Children's Hospital, University of Cologne, Cologne, Germany; 11 University Children's Hospital, Albert Ludwigs University, Freiburg, Germany; 12 Kinder- und Jugendarztpraxis Laub, Rosenheim, Germany; 13 Children's Department, University Teaching Hospital, Landeskrankenhaus Feldkirch, Feldkirch, Austria; 14 University Children's Hospital Vienna, Vienna, Austria; 15 Institute of Epidemiology, Ulm University, Ulm, Germany; 16 Centre National de Génotypage, Institut Génomique, Commissariat à l'Énergie Atomique, Evry, France; 17 Centro Nacional de Analisis Genomico, Barcelona, Spain; Innsbruck Medical University, Austria

## Abstract

The first genome wide association study (GWAS) for childhood asthma identified a novel major susceptibility locus on chromosome 17q21 harboring the *ORMDL3* gene, but the role of previous asthma candidate genes was not specifically analyzed in this GWAS. We systematically identified 89 SNPs in 14 candidate genes previously associated with asthma in >3 independent study populations. We re-genotyped 39 SNPs in these genes not covered by GWAS performed in 703 asthmatics and 658 reference children. Genotyping data were compared to imputation data derived from Illumina HumanHap300 chip genotyping. Results were combined to analyze 566 SNPs covering all 14 candidate gene loci. Genotyped polymorphisms in *ADAM33*, *GSTP1* and *VDR* showed effects with p-values <0.0035 (corrected for multiple testing). Combining genotyping and imputation, polymorphisms in *DPP10*, *EDN1*, *IL12B*, *IL13*, *IL4*, *IL4R* and *TNF* showed associations at a significance level between p = 0.05 and p = 0.0035. These data indicate that (a) GWAS coverage is insufficient for many asthma candidate genes, (b) imputation based on these data is reliable but incomplete, and (c) SNPs in three previously identified asthma candidate genes replicate in our GWAS population with significance after correction for multiple testing in 14 genes.

## Introduction

Genome wide association studies (GWAS) have recently identified genetic susceptibility for many complex diseases by genotyping hundreds of thousands of single nucleotide polymorphisms (SNPs) across the genome in very large sets of patients and controls [1]. While GWAS discovered novel disease loci, many previous candidate genes identified by hypothesis driven approaches were not replicated by GWAS at a genome wide significance level. Thus, the question arose in many complex disorders if associations with previous candidate genes represent false positive results. Alternatively it was suggested that GWAS SNP chips insufficiently covered these genes of interest. Imputation was recently introduced to predict allelic status of SNPs not covered by direct genotyping in GWAS, extrapolating information from neighbouring SNPs for which genotyping data and linkage disequilibrium (LD) from HapMap was available. The value of these approaches is not yet determined. We used our GWAS data set on asthma [2] to study these open questions specifically for candidate genes in asthma.

In childhood asthma, the first GWAS [2] to which we contributed a discovery population of app. 1.400 German children, captured a major susceptibility signal from chromosome 17q21 and a few less suggestive hits in other regions of the genome, none of which was overlapping with the numerous asthma candidate genes previously identified by hypothesis driven approaches or positional cloning [3].

Therefore, we developed bioinformatics tools to systematically identify asthma candidate genes from the literature and genotyped these genes for previously suggested asthma related SNPs. We compared these results with the initial GWAS genotyping and imputation data derived from our original GWAS genotyping [2]. We used the combination of these approaches to cover all candidate genes in depth with tagging SNPs and performed a comprehensive analysis of previous candidate gene loci.

## Results

**Figure 1:**
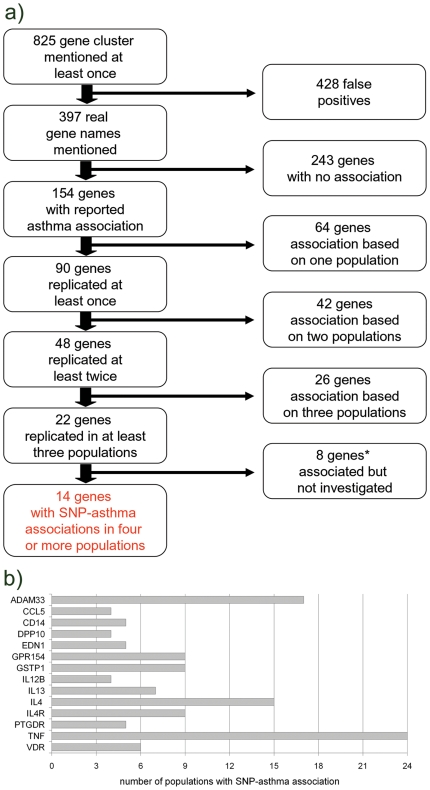
**a) Schematic depiction of literature query process.** * genes not further investigated were (a) *ORMDL3*, originally discovered in this GWA; (b) discovered by other GWAS: *CHI3L1*, *IL1RL1*, *MYB*, *WDR36* (c) *NOS1* where CA-repeats and not SNPs were reported; (d) gene deletions for *GSTM1*, *FLG.* **b) Number of populations that show an association between SNPs in the 14 selected candidate genes and asthma diagnosis.** (based on literature query from October 22^nd^ 2009).

We implemented a script to scan an assemblage of abstracts for specific gene names or collections of gene names as depicted in figure 1a. The script searches for single words or specific groups of words in a given text. Gene names or a group of several names for the same gene were searched in a collection of abstracts. For every gene name or cluster of gene names respectively a separate file was produced that consists of all abstracts a gene of the cluster is mentioned in. Gene names and symbols were obtained from the Human Genome Organization - Gene Nomenclature Committee [4]. We scanned all 28,472 approved symbols in the HUGO database as of October 22^nd^ 2009. If available we clustered these gene names with their previous symbols and their attached aliases so that overall 98743 specific gene names were searched against a selection of abstracts published in the pubmed database (http://www.pubmedcentral.nih.gov/) as of October 22^nd^ 2009. For the current study we searched for the terms “asthma” and “association” and “SNP” or “polymorphism” or “genetic”, retrieving 1,341 abstracts. With the script 825 cluster of gene names, hence genes, were extracted. Details on the use of the bioinformatics search tool for genetic association data from the literature are available upon request from the authors. All abstracts were then examined to contain significant associations (nominal p-value of <0.05) with asthma. For that purpose, different forms of asthma like childhood, adult or atopic asthma were not discriminated and reports were not weighted for study size. Associations with asthma severity or intermediate phenotypes of asthma were not considered and the analysis was restricted to studies in English language. All genes for which genetic associations with asthma were reported at the gene level in a discovery dataset and at least three independent populations (not necessarily published independently) were studied further. Direction of association and associated SNP in a gene could vary (loose replication) as indicated in table 1. Genes discovered by further GWAS studies [5], [6] were excluded in this study as their discovery relied on SNPs present at currently used SNP chips.

**Table 1:** Comparison of risk allele, frequency of risk allele in cases and controls and effect size in our study population and previously published studies for candidate gene polymorphisms with a p- value of <0.05 in our population (for complete results on all tested SNPSs see table S2).

current study				summary of previous published results		
gene & SNP rs nr	risk allele	freq cases/controls	Odds ratio (95%CI)	study, reference, origin, and n of cases (freq cases/freq controls)	risk allele	odds ratio (95%CI)
ADAM33 rs528557	G	0.29/0.22	1.51 (1.25–1.82)	Thongngarm et al. ^[19]^ + Thai n = 200 (0.24/0.18)	C	1.94 (1.16–3.26)
				Eerdewegh et al.^[20]^ + Caucasian n = 130 (0.84/0.73)	G	1.93 (1.08–3.56)
				Howard et al.^[21]^ Hispanic n = 111 (0.23/0.17)	C	1.38 (1.00–3.23)
				Howard/Afric Amer n = 157 (0.70/0.63)	C	1.79 (1.04–2.44)
				Hirota et al. ^[22]^ + Japanese n = 482 (0.79/0.75)	G	1.25 (1.02–1.54)
				Blakey et al. ^[23]^ British n = 1306 (0.75/0.73)	C	1.09 (1.01–1.16)
EDN1 rs5369	G	0.89/0.86	1.36 (1.04–1.72)	Zhu et al. ^[24]^ Norwegian n = 267 (0.11++)	A	A risk allele*
EDN1 rs1629862	G	0.89/0.86	1.32 (1.04–1.64)	Zhu/British n = 907 (0.90++)	G	G risk allele*
				Zhu/Norwegian n = 267 (0.90++)	G	G risk allele*
IL13 rs20541	A	0.24/0.21	1.23 (1.03–1.48)	Black et al.^[25]^ + British n = 275 (0.22/0.17)	A	1.39 (1.11–1.73)
				Heinzmann et al.^[26]^ Japanese n = 100 (0.63/0.43)	A	1.81 (1.11–2.93)
				Heinzmann/British n = 150 (0.40/0.27)	A	2.14 (1.28–3.60)
				Hosseini-Farahabadi et al.^[27]^ + Iranian n = 30 (0.41/0.22)	A	2.52 (1.19–5.40)
IL4R rs2057768	T	0.30/0.25	1.27 (1.06–1.51)	Hytonen et al. ^[28]^ Swedish n = 170 (0.21++)	T	T risk haplo**
IL4R rs1805010	G	0.48/0.43	1.23 (1.05–1.45)	Zhang et al. ^[29]^ Chinese n = 352 +++ (0.46/0.38)	A	1.38 (1.01–1.88)
				Beghe et al. ^[30]^ British = 326 (0.45++)	A	A risk haplo**
				Zhang et al. ^[31]^ Chinese n = 145 (0.49++)	G	G risk haplo**
				Zhang/Malasian n = 73 (0.47++)	G	G risk haplo**
				Ober et al. ^[32]^ Hutterite n = 77 (0.48++)	G	G risk haplo**
				Hytonen et al. ^[28]^ Swedish n = 170 (0.47++)	G	G risk haplo**
TNF rs1800629	A	0.18/0.14	1.34 (1.08–1.66)	Li et al. ^[33]^ Californian n = 403 (0.13++)	A	1.25 (1.11–1.43)
				Castro-Giner et al.^[34]^ European n = 278 (0.21/0.15)	A	1.49 (1.22–1.81)
				Wu et al. ^[35]^ Mexican n = 596 (0.05/NA)	A	1.54 (1.04–2.28)
				Munthe-Kaas et al. ^[36]^ Norwegian n = 268 (NA)	A	1.6 (1.2–2.0)
				Kim et al. ^[37]^ Korean n = 719 (0.07/0.04)	A	1.77 (1.05–2.82)
				Kumar et al. ^[38]^ + Indian n = 155 (0.14/0.08	A	1.79 (1.01–2.98)
				Witte et al. ^[39]^ mixed American n = 236 (0.16/0.13)	A	1.86 (1.03–3.34)
				Kumar et al ^[40]^ Indian n = 123 (0.16/0.09)	A	1.9 (1.31–2.49)
				Wang et al. ^[41]^ Taiwanese n = 140 (0.15/0.07)	A	2.17 (1.29–4.33)
				Jiménez-Morales et al ^[42]^ Mexican n = 226 (0.06/0.03)	A	2.32 (1.28–4.20)
				Winchester et al.^[43]^ British/Irish n = 20 (0.43/0.18)	A	2.6 (1.1–6.2)
				Chagani et al. ^[44]^ Caucasian n = 251 (NA)	A	3.07 (1.1–5.5)
				Moffat et al. ^[45]^ Australian n = 92 (0.3/0.18)	A	3.57 (2,17–10.42)
				Shin et al. ^[46]^ Korean n = 550 (0.95/0.88)	G	2.7 (1.56–4.76)
				Albuquerque et al. ^[47]^ Caucasian n = 74 (0.83/0.77)	G	2.38 (1.3–4.55)
VDR rs1540339	G	0.65/0.59	1.28 (1.10–1.49)	Poon et al. ^[48]^ Quebecian n = 347 (0.4++)	A	A risk allele*
VDR rs3782905	C	0.32/0.28	1.21 (1.02–1.44)	Raby et al. ^[49]^ + European ancestry n = 517 (0.33/0.28)	C	1.26 (1.03–1.53)
				Poon et al. ^[48]^ Quebecian n = 347 (0.31++)	C	C risk allele*

+ The OR and confidence intervals were calculated based on published data.

++ Allele frequency of the entire study is provided if separate allele frequencies were not applied or haplotype or family studies have been conducted.

+++ In the Chinese population the G allele is the major allele.

*Allele is either announced as the risk allele or described to be overtransmitted to asthmatic patients.

**The haplotype containing this allele is associated with the susceptibility to asthma.

The most prevalent gene symbol in the retrieved abstracts for genetic association with asthma was *IL4* (91 citations) followed by *IL13* (75 citations) and *TNF* (73 citations). Significant associations of SNPs in *IL4* with asthma were reported in 15 different populations. The following 14 genes distributed across the genome were associated with asthma diagnosis in more than three independent study populations (figure 1b): *ADAM33*, *CCL5*, *CD14*, *DPP10*, *EDN1*, *GPR154*, *GSTP1*, *IL12B*, *IL13*, *IL4*, *IL4R*, *PTGDR*, *TNF*, and *VDR*.

In these genes, asthma association had been reported for a total of 89 SNPs (table 2 and table S1); only 19 of these had been directly genotyped by the Illumina Sentrix HumanHap300 BeadChip used in our GWAS. Using linkage disequilibrium (LD) based on HapMap [7], a further 14 of these 89 SNPs were indirectly covered by tagging SNPs from chip-genotyping. Thus, only a total of approximately 37% of previously suggested SNPs in asthma candidate genes were captured directly or indirectly by GWAS genotyping. None of the SNPs in *ADAM33*, *CCL5*, *CD14*, or *IL4* (table 2) were covered by the original GWAS genotyping.

**Table 2:** Number of previously associated asthma candidate gene SNPs and coverage by 1^st^ generation GWAS genotyping, imputation and re-genotyping.

gene	chromosome	N of SNPs with reported association	GWAS genotyping * (genotyped directly/tagged indirectly)	imputation HapMapII ** (genotyped directly/tagged indirectly)	imputation HapMap III ** (genotyped directly/tagged indirectly)	re-genotyped ***
ADAM33	20p13	18	0/0	6/0	6/0	14[2]
CCL5	17q11	2	0/0	1/0	1/0	2
CD14	5q31	2	0/0	2/0	1/0	1
DPP10	2q12	5	0/4	5/0	3/0	0
EDN1	6p24	9	2/3	6/1	5/0	0[2]
GPR154	7p15	12	3/2	9/0	4/0	5[1]
GSTP1	11q13	2	1/0	1/0	1/0	1
IL12B	5q31	5	2/0	5/0	4/0	1
IL13	5q31	3	1/0	1/0	2/0	2
IL4	5q31	3	0/0	2/0	3/0	2
IL4R	16p12	8	4/1	6/0	7/0	2[1]
PTGDR	14q22	6	1/2	2/1	2/0	3
TNF	6p21	5	0/1	3/0	3/0	3[1]
VDR	12q12	9	5/1	7/0	7/0	3
sum of SNPs		89	33 (19/14)	58 (56/2)	49	39[7]

*on Illumina Sentrix HumanHap300 BeadChip.

**based on Illumina Sentrix HumanHap300 BeadChip and Hapmap phase II or III respectively.

***by MALDI-TOF or TAQMAN.

[ ] indicates SNPs for which genotyping failed.

We aimed at re-genotyping all additional candidate gene SNPs and performed imputation based on GWAS genotyping. From 56 SNPs not covered be GWAS genotyping, genotyping assays could be designed for 45 of which 39 passed quality control. For 58 of the 89 candidate-gene derived SNP imputation was successful. Thus, for 11 of the 89 SNPs only imputation but no genotyping data and for 55 candidate SNPs (with previous association) imputation and genotyping data were available. In cases where both imputation and genotyping results were available for a specific SNP, genotyping results were always used for further analyses. Using GWAS genotyping data, re-genotyping and imputation in combination, data for 83 out of 89 candidate gene SNPs from the literature could be analyzed successfully.

SNPs in the genes *ADAM33* (rs528557, p = 0.000019) and *VDR* (rs1540339, p = 0.0014), previously associated with asthma, were also associated with asthma in this dataset at a significance level of 0.0035 corrected for multiple testing. Previously associated SNPs in *EDN1*, *IL13*, *IL4R* and *TNF* replicated at a nominal significance level between 0.05≥p>0.0035. Effect size and effect directions were compared to previously published results in table 1.

**Figure 2:**
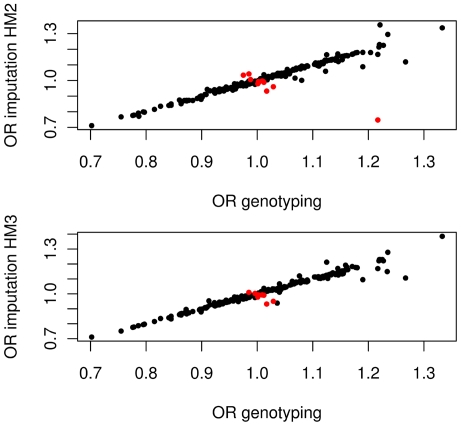
Correlation between effect sizes observed by concomitant genotyping and imputation of candidate gene SNPs for which both measures were available. (red indicates change of effect direction between genotyping and imputation). For the comparison based on HapMapII (HM2) data 241 SNPs and for HapMapIII (HM3) 216 SNPs were available.

**Figure 3:**
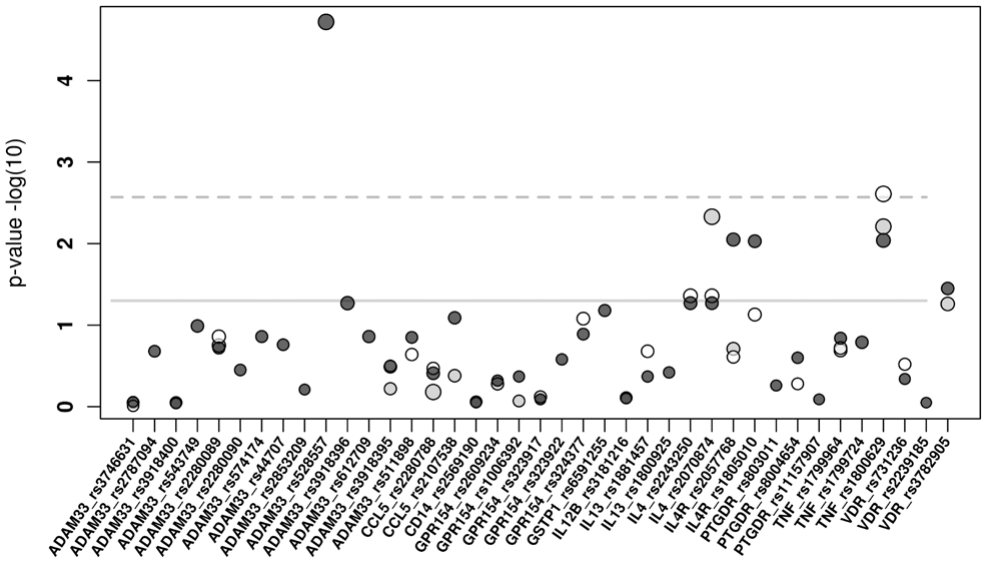
Comparison of effect sizes and p-values derived from regenotyped SNPs with data based on imputation. Log transferred p-values are shown on the y-axis. Each individual SNP effect is depicted as a circle where the diameter of the circle reflects the respective odds ratios. The dark grey circles represent the regenotyping the light grey circles stand for the imputation based on HapMap2 and the transparent show the imputation data based on HapMap3. Significance level are marked as solid (p = 0.05) and dashed grey lines (p = 0.0035).

For 241 SNPs in the 14 candidate genes both imputation and direct genotyping data were available irrespective of previously reported associations (table S2). Effect size and p-value of effects correlated tightly (figure 2), and R^2^ is given in table S2. Direction of effects changed 12 times, mostly when effects were close to 1. However, in one case (rs2280788, *CCL5*) imputation implied an odds ratio (OR) of 0.75 when re-genotyping showed an effect size of 1.22. For *IL4*, imputation suggested rs2070874 (C-33T) to be significantly associated with asthma (p = 0.005) but the association was not significant when re-genotyped by matrix-assisted laser desorption/ionization time-of flight (MALDI-TOF) (p = 0.054). An opposite outcome was observed for *IL4R* rs2057768, when an effect was significant by genotyping but not by imputation (p = 0.009 versus p = 0.2). This suggests that imputation is a reasonably reliable tool to estimate SNP effects of asthma candidate genes based on 1^st^ generation GWAS genotyping data, when effect sizes are beyond 1.25 in a population of app. 1,000 cases. For detailed comparison between re-genotyped and inputated SNPs see figure 3. However, imputation based on GWAS BeadChip genotyping data could only increase coverage of the previously reported SNPs in asthma candidate genes to app. 60%; missing numerous SNPs of specific interest.

**Figure 4:**
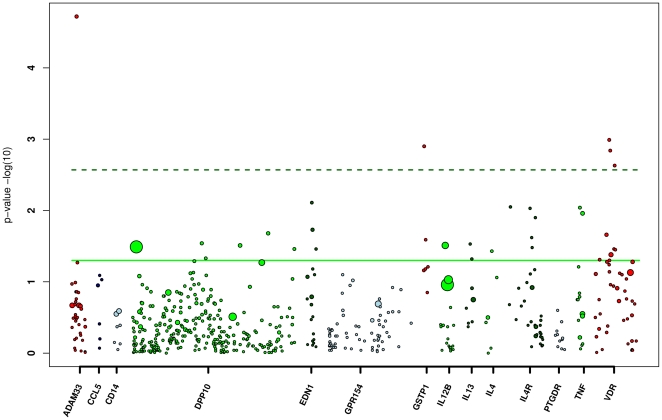
Association of 566 tagging SNPs in 14 candidate genes with asthma. Genes are positioned along the x-axis in alphabetical order. Each dot represents SNPs in their relative position within the gene. Log transferred p-values are shown on the y-axis. Each individual SNP effect is depicted as a circle where the diameter of the circle reflects the respective odds ratios. Significance level are marked as solid (p = 0.05) and dashed green lines (p = 0.0035).

Thus, we next combined data from 1^st^ generation GWAS genotyping by BeadChip, re-genotyping by MALDI-TOF and TaqMan® MGB, and imputation based on this combined set of genotypes to comprehensively saturate all 14 candidate gene loci under study with 566 tagging SNPs as well as previously associated SNPs (figure 4 and table S2). This way, further associations below the 0.0035 threshold were detected for SNPs in *GSTP1* and VDR. Further SNPs in *DPP10*, *IL12B* and *IL4* were now found to be associated with asthma in our population, but only at the nominal significance level of 0.05 (table S2).

## Discussion

We systematically investigated the role of SNPs from previously suggested asthma candidate genes with more than three positive association reports in the literature. In a large German population for which 1^st^ generation GWAS was also available, imputation was provided based on Illumina Sentrix HumanHap300 BeadChip data and compared to re-genotyping of candidate gene SNPs by MALDI-TOF and TaqMan® techniques. Thus, an in depth analysis of these previously suggested candidate genes was performed. The study was powered to detect asthma odds ratios of 1.5 at a significance level of alpha = 0.05 with a power of 95% for SNPs with a minor allele frequency of >0.1 and for odds ratios of 1.3 with a power of 80%. SNPs in three of 14 investigated candidate genes showed associations at a significance level corrected for multiple testing (p<0.0035), and further 7 genes were associated at a significance level of p<0.05. Effect directions were consistent for associated SNPs in our population compared to previous publications in populations of similar ancestry (table 1).

We expected to observe less than five positive associations at a nominal significance level of p<0.05 under the null hypothesis of no association (83×0.05 = 4.15). We observed 9 SNPs associated with asthma at this level, twice more than expected by chance. We corrected for multiple testing by multiplying the nominal significance level of alpha = 0.05 by the number of genes studied (N = 14). This seems appropriate for the purpose of replication testing. When extending the analyses to all 566 SNPs in the respective gene locus, this level of correction may be too permissive. Also, one has to keep in mind that novel associations found with the broader approach of saturating tagging SNPs lack the level of replication which the SNPs drawn from the literature have already achieved. Therefore, the associations found with rs614080 and rs6591256 in *GSTP1*, rs17764121, rs1396936, rs966859, rs17823623, rs12997234 and rs13034936 in *DPP10*, rs3213102 in *IL12B* and rs2243274 in *IL4* need to be treated with caution until a sufficient level of replication has been achieved for these SNPs in other populations.

Imputation was compared to re-genotyping in our dataset to assess the usefulness of imputation to answer such a specific question as to what extent certain candidate gene SNPs play a role in asthma in a GWAS data set. Clearly, accuracy of imputation depends on coverage of the specific locus under investigation. With 1^st^ generation GWAS SNP chips, gray and black areas with low or no SNP coverage exist in the genome (table S3). Interestingly, this is also the case for well known genes associated with many diseases: For *CD14* and *IL4*, no SNP was genotyped by the Illumina Sentrix HumanHap300 BeadChip. This has improved for later versions of GWAS genotyping chips (table S3). But still, the coverage of putatively functional SNPs previously associated with disease is insufficient for many genes even in next generation GWAS chips and even when tagging SNP approaches based on LD were taken into account (table S3).

LD for our imputation was derived from HapMap2 version 2006 as well as HapMap3 version 2009. With further improvements of HapMap and the finalisation of the 1000 Genomes project, where 1000 human genomes are currently being sequenced (www.1000genomes.org), imputation information also on existing SNP sets will improve and re-analysis of existing GWAS data may be useful. Despite it's known limitations, imputation proved valuable and helpful in this project. Reliability of results was satisfactory when compared to real genotyping results in those cases where information from both sources were acquired. However, re-genotyping of major susceptibility signals found by imputation may still be useful as this represents the gold standard for ascertainment of allelic status of SNPs.

Effects of candidate gene SNPs on asthma risk are modest, not exceeding a change in risk by more than app. 50% (odds ratios between 0.58 and 1.50) which is within the expected range for genetic effects in complex diseases and not significantly different from susceptibility signals reported by GWAS in atopic diseases [8]–[10]: The chromosome 17q21 susceptibility locus shows an asthma effect size of OR 1.11–1.52 [2], and a further GWAS on asthma report ORs of 0.88 respectively [9]. Odds ratios for asthma identified through intermediate phenotypes by GWAS were 0.54 [5] and 1.07–1.16 [6]. A recently reported susceptibility locus for atopic dermatitis on 11q13.5 shows an overall effect of OR 1.22 [8]. These data indicate that a number of previously suggested asthma candidate gene SNPs influence asthma susceptibility at a modest level, comparable to those genes identified through GWAS in allergy genetics. Effects can be detected in large study populations but effect size may be minor and significance of effects and effect direction may vary in smaller studies or be influenced by environmental factors in specific populations (table 1). Highly significant p-values of associations in initial GWAS reports may imply but not guarantee replication of associations in further cohorts. At the same time, biological candidates such as those identified through hypothesis driven candidate gene approaches may not yield p-values significant after correction for multiple testing in GWAS but may replicate consistently over many populations tested. In some cases, as shown in table 1, replication of gene effects on asthma is on a gene level rather than on the individual SNP level, indicating either that the putatively causal SNP may not be typed or that gene by environment effects may modify effects; definitions of asthma phenotypes may vary between studies and different genetic models may have been applied in different studies [11]. Publication bias may be present for candidate genes and could have influenced the selection of genes exceeding the threshold of 3 positive replications. For all mentioned genes and susceptibility signals, systematic replications are thus required before final conclusions can be drawn. Taken all considerations into account, the strongest evidence for true association between a previous candidate SNP and asthma in this population exists for rs528557 in ADAM33, while the signal from chromosome 17q21 is the most replicated finding from GWAS studies so far.

Our analyses suggest that GWAS is a powerful tool to discover novel susceptibility loci for complex diseases and it has recently been applied very successfully. However, GWAS have limitations that may be overcome with improvements in SNP coverage and imputation. At this point GWAS are not designed to finally exclude potential candidate genes from a role in a specific disease. This is particularly the case for complex diseases where a single study can not cover all potential phenotypes, all possible gene-environment interactions and all different genetic models. Replication in as many populations as possible is still important, as well as the determination of biological function for candidate genes and SNPs in a specific disease. This is the challenge for the post-GWAS era.

## Methods

### Population description

**Figure 5:**
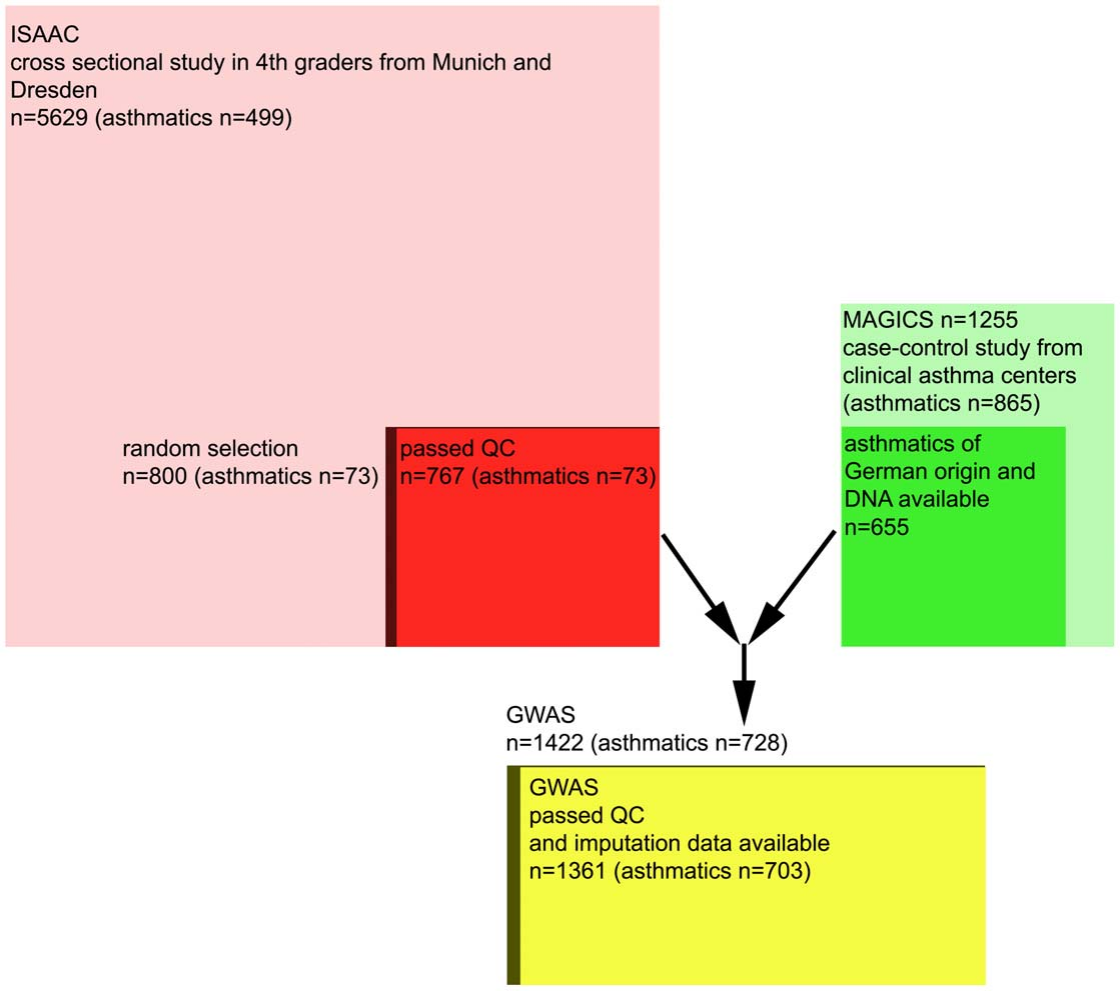
Study Design and population selection strategy. (ISAAC  =  International Study for Asthma and Allergies in Childhood; MAGICS  =  Multicentre Asthma Genetics in Childhood Study; GWAS  =  Genome wide Association Study; QC  =  quality control).

An overview of the population selection for this study is given in figure 5 and described briefly here. From 2001 to 2007, the Multicenter Asthma Genetic in Childhood (MAGIC) study recruited 1255 children in seven German and Austrian clinical asthma centers located in Bochum, Cologne, Feldkirch, Freiburg, Munich/Rosenheim, Vienna and Wesel. A pediatric pulmonologist diagnosed asthma and allergies according to clinical guidelines and objective measures such as lung function tests, clinical examination, history, and allergy testing in 865 children (mean age 11 years, range 3–17 years, 64% male). Children without asthma but other conditions (such as inflammatory bowel disease) were also recruited in another leg of the study in the same clinical centers (n = 390) but have not been included in any analysis so far. All 655 children from the MAGIC study that were included in this and previous analyses [2] were diagnosed with asthma, were of German origin and had sufficient high quality DNA available at the time the genome wide analysis was performed in 2006.

As a reference, a random sample consisting of 800 German children was drawn from the cross sectional International Study of Asthma and Allergies in Childhood phase II (ISAAC II). 767 passed DNA quality control for GWAS and of these, 73 were asthmatics and 694 were non-asthmatics. The ISAAC II population had been described in great detail before. In brief, the study assessed the prevalence of asthma and allergies in schoolchildren aged 9–11 years in Munich and Dresden in 1995 and 1996 [12]. Asthma status was based on the parent's report of a physician's diagnosis of asthma at least once, or of spastic or asthmatic bronchitis more than once in self-administered questionnaires.

Written informed consent was obtained from all parents of children included in these studies and study methods were approved by the respective ethics committees. All children were of German origin to control for population admixture [2].

### Genotyping

Technical details on GWAS genotyping of our study population using the Illumina Sentrix HumanHap300 BeadChip have been published elsewhere [2]. For additional in depth re-genotyping of candidate gene SNPs, matrix-assisted laser desorption/ionization time-of flight (MALDI-TOF) mass spectrometry (Sequenom Inc., San Diego, USA) was used as previously described [13]. SNPs failing MALDI-TOF genotyping were genotyped by a TaqMan® MGB biallelic discrimination system as previously described [14]. Deviations from Hardy–Weinberg Equilibrium (HWE) in the control group were assessed for quality control of genotyping procedures. Detailed information including specific amplification and extension primers for all genotyping procedures are provided by the authors upon request.

### Statistical analyses and quality control

Statistical analyses were performed with the PLINK [15] software package (version 1.04, http://pngu.mgh.harvard.edu/purcell/plink/) and confirmed with the SAS statistical software package (version 9.2; SAS, Inc., Cary, NC). All associations of asthma with SNPs, their accompanying odds ratio and confidence intervals were evaluated by the additive logistic regression model. We restricted the analysis to one genetic model (which is the common model for GWAS) to reduce the problem of multiple testing. Power calculations were performed with the PGA software package [16].

We imputed genotypes for all polymorphic HapMap SNPs (www.hapmap.org) by using a hidden Markov model programmed in MACH [17]. The method combines genotypes from the study samples with the HapMap CEU sample (July 2006 phased haplotype release, called HapMapII and Feb 2009 phased haplotype release, called HapMapIII) and identifies the stretches of haplotype shared between the study samples and the HapMap sample. For each individual, the genotype at the untyped SNP can be summarized by taking (1) the most likely genotype according to the posterior probability of the three possible genotypes and (2) allele dosage, the expected number of copies of the reference allele (a fractional value between 0 and 2). We used the imputed most likely genotypes for association analyses.

Exclusion criteria for further analyses were a low genotyping rate (<95%) with the Illumina Sentrix HumanHap300 BeadChip, incomplete phenotype information, unusual high heterozygosity, missing age information or age above 18 years, genetic identification of cryptic cousin pairs or non-Caucasian origin. Thus, for further analyses 703 asthmatics, of which 414 were males and 658 controls (372 males), remained. Table 3 provides additional clinical information. Background stratification was measured by a genomic control parameter of 1.01. All markers which failed the HWE test in controls (p< = 0.0001), had low minor-allele frequencies (<5%) or low genotyping call rates (<95%) were also excluded. The remaining 300,745 SNPs were used for further analyses. For re-genotyping of candidate gene SNPs in children with sufficient DNA available, genotype call rates of at least 95% were achieved by MALDI-TOF and TaqMan® MGB assays. The software Haploview [18] was used to calculate linkage disequilibrium (LD) patterns and to select tagging SNPs (MAF 0.001, r2>0.8). The chi-square test was applied to analyse deviations from Hardy-Weinberg equilibrium (HWE), with expected frequencies derived from allele frequencies. The alpha level was set to 0.0035 to correct for multiple testing of 14 genes (0.05/14 = 0.0035).

**Table 3:** Characteristics of the population used for the actual study consisting of two parts, the Multicentre Asthma Genetics in Childhood Study (MAGICS) and the International Study for Asthma and Allergies in Childhood (ISAAC).

	MAGICS		ISAAC	
	No.*	percentage	No.*	percentage
male sex	414/631	65.6	372/730	51
age, mean (SD)	10.97 (2.92)		9.62 (0.78)	
asthmatic	631/631	100	72/730	9.9
atopic sensitization	498/617	80.9	302/729	41.4

*Number affected/number with data available.

## Supporting Information

Table S1 Gene symbol, references, total number of SNPs and individual rs numbers of asthma associated SNPs
(0.28 MB DOC)

Table S2 Candidate gene associations with asthma and comparison between genotyping and imputation results. Odds ratios, 95% confidence intervals are given for SNP effects. R2 is given for SNPs were both imputation and genotyping data is available. Bold font indicates previously reported SNPs, standard font is tagging SNPs, cursive font indicates SNPs that are neither tagging nor previously reported SNPs; * p<0.05; **p<0.0035
(0.10 MB PDF)

Table S3 Comparison of candidate gene SNP coverage by GWAS genotyping systems. Number of genotyped SNPs per candidate gene locus from 5 kb upstream of 1st exon to 5 kb after last exon are given and in brackets () genotyped or covered SNPs with previously reported asthma association.
(0.04 MB DOC)
