# Supplementary material for: Unifying Candidate Gene and GWAS Approaches in Asthma
Source: PLoS One. 2010 Nov 12;5(11):e13894. doi: 10.1371/journal.pone.0013894 (PMC2980484; doi:10.1371/journal.pone.0013894)
Supplement: Table S1 — Gene symbol, references, total number of SNPs and individual rs numbers of asthma associated SNPs (0.28 MB DOC) [file pone.0013894.s001.doc]

**Supplementary table S**1

| gene | N of SNPs with reported association | rs numbers of SNPs with reported association  (* genotyping failed) |
| --- | --- | --- |
| ADAM33 [1-12] | 18 | rs3746631, rs2787094, rs3918400, rs543749, rs628977*, rs2280089, rs2280090, rs2280091, rs574174, rs44707, rs2853209, rs528557, rs3918396, rs612709, rs918395, rs2280092*, rs511898, rs598418 |
| CCL5 [13-16] | 2 | rs2107538, rs2280788 |
| CD14 [17-21] | 2 | rs2569190, rs4914 |
| DPP10 [3,22,23] | 5 | rs13392783, rs13011555, rs843385, rs7576583, rs272071 |
| EDN1 [24-26] | 9 | rs1800541*, rs1476046, rs1800543, rs5369, rs1629862, rs5370, rs2071942*, rs1630736, rs9369217 |
| GPR154 [22,25,27-30] | 12 | rs2609234, rs1006392, rs714588, rs1379928, rs963218, rs2609215, rs323917, rs740347, rs324396, rs323922, rs324377, rs324384* |
| GSTP1 [31-38] | 2 | rs6591255, rs1695 |
| IL12B [39-41] | 5 | rs3212227, rs2853694, rs2569254, rs3181216, rs2546890 |
| IL13 [25,42-48] | 3 | rs1881457, rs1800925, rs20541 |
| IL4 [44,49-61] | 3 | rs2243250, rs2070874, rs2243248 |
| IL4R [54,60,62-66] | 8 | rs2057768, rs1805010, rs2234898, rs1805011, rs1805012, rs2234900*, rs1805015, rs1801275 |
| PTGDR [67-69] | 6 | rs803011, rs8004654, rs803010, rs11157907, rs708486, rs708487 |
| TNF [42,58,70-89] | 5 | rs1800630, rs1799964, rs1799724, rs1800629, rs361525* |
| VDR [25,90-92] | 9 | rs7975232, rs731236, rs1544410, rs2239185, rs2239182, rs1540339, rs3782905, rs2853564, rs2239186 |

**References supplement**

1. Van Eerdewegh P, Little RD, Dupuis J, Del Mastro RG, Falls K, et al. (2002) Association of the ADAM33 gene with asthma and bronchial hyperresponsiveness. Nature 418: 426-430.

2. Thongngarm T, Jameekornrak A, Limwongse C, Sangasapaviliya A, Jirapongsananuruk O, et al. (2008) Association between ADAM33 polymorphisms and asthma in a Thai population. Asian Pac J Allergy Immunol 26: 205-211.

3. Blakey JD, Sayers I, Ring SM, Strachan DP, Hall IP (2009) Positionally cloned asthma susceptibility gene polymorphisms and disease risk in the British 1958 Birth Cohort. Thorax 64: 381-387.

4. Su D, Zhang X, Sui H, Lu F, Jin L, et al. (2008) Association of ADAM33 gene polymorphisms with adult allergic asthma and rhinitis in a Chinese Han population. BMC Med Genet 9: 82.

5. Howard TD, Postma DS, Jongepier H, Moore WC, Koppelman GH, et al. (2003) Association of a disintegrin and metalloprotease 33 (ADAM33) gene with asthma in ethnically diverse populations. J Allergy Clin Immunol 112: 717-722.

6. Werner M, Herbon N, Gohlke H, Altmuller J, Knapp M, et al. (2004) Asthma is associated with single-nucleotide polymorphisms in ADAM33. Clin Exp Allergy 34: 26-31.

7. Raby BA, Silverman EK, Kwiatkowski DJ, Lange C, Lazarus R, et al. (2004) ADAM33 polymorphisms and phenotype associations in childhood asthma. J Allergy Clin Immunol 113: 1071-1078.

8. Kedda MA, Duffy DL, Bradley B, O'Hehir RE, Thompson PJ (2006) ADAM33 haplotypes are associated with asthma in a large Australian population. Eur J Hum Genet 14: 1027-1036.

9. Qiu YM, Luo YL, Lai WY, Qiu SJ (2007) [Association between ADAM33 gene polymorphism and bronchial asthma in South China Han population]. Nan Fang Yi Ke Da Xue Xue Bao 27: 485-487.

10. Qiu YM, Luo YL, Lai WY, Qiu SJ, Wu YX (2007) [Association of polymorphism of Met764Thr locus allele in ADAM33 gene with bronchial asthma and lung function of asthmatic subjects]. Zhonghua Jie He He Hu Xi Za Zhi 30: 518-521.

11. Noguchi E, Ohtsuki Y, Tokunaga K, Yamaoka-Sageshima M, Ichikawa K, et al. (2006) ADAM33 polymorphisms are associated with asthma susceptibility in a Japanese population. Clin Exp Allergy 36: 602-608.

12. Hirota T, Hasegawa K, Obara K, Matsuda A, Akahoshi M, et al. (2006) Association between ADAM33 polymorphisms and adult asthma in the Japanese population. Clin Exp Allergy 36: 884-891.

13. Fryer AA, Spiteri MA, Bianco A, Hepple M, Jones PW, et al. (2000) The -403 G-->A promoter polymorphism in the RANTES gene is associated with atopy and asthma. Genes Immun 1: 509-514.

14. Yao TC, Kuo ML, See LC, Chen LC, Yan DC, et al. (2003) The RANTES promoter polymorphism: a genetic risk factor for near-fatal asthma in Chinese children. J Allergy Clin Immunol 111: 1285-1292.

15. Al-Abdulhadi SA, Helms PJ, Main M, Smith O, Christie G (2005) Preferential transmission and association of the -403 G --> A promoter RANTES polymorphism with atopic asthma. Genes Immun 6: 24-30.

16. Lachheb J, Chelbi H, Hamzaoui K, Hamzaoui A (2007) Association between RANTES polymorphisms and asthma severity among Tunisian children. Hum Immunol 68: 675-680.

17. Sharma M, Batra J, Mabalirajan U, Goswami S, Ganguly D, et al. (2004) Suggestive evidence of association of C-159T functional polymorphism of the CD14 gene with atopic asthma in northern and northwestern Indian populations. Immunogenetics 56: 544-547.

18. Tremblay K, Daley D, Chamberland A, Lemire M, Montpetit A, et al. (2008) Genetic variation in immune signaling genes differentially expressed in asthmatic lung tissues. J Allergy Clin Immunol 122: 529-536 e517.

19. Smit LA, Siroux V, Bouzigon E, Oryszczyn MP, Lathrop M, et al. (2009) CD14 and toll-like receptor gene polymorphisms, country living, and asthma in adults. Am J Respir Crit Care Med 179: 363-368.

20. Zambelli-Weiner A, Ehrlich E, Stockton ML, Grant AV, Zhang S, et al. (2005) Evaluation of the CD14/-260 polymorphism and house dust endotoxin exposure in the Barbados Asthma Genetics Study. J Allergy Clin Immunol 115: 1203-1209.

21. Lachheb J, Dhifallah IB, Chelbi H, Hamzaoui K, Hamzaoui A (2008) Toll-like receptors and CD14 genes polymorphisms and susceptibility to asthma in Tunisian children. Tissue Antigens 71: 417-425.

22. Hersh CP, Raby BA, Soto-Quiros ME, Murphy AJ, Avila L, et al. (2007) Comprehensive testing of positionally cloned asthma genes in two populations. Am J Respir Crit Care Med 176: 849-857.

23. Allen M, Heinzmann A, Noguchi E, Abecasis G, Broxholme J, et al. (2003) Positional cloning of a novel gene influencing asthma from chromosome 2q14. Nat Genet 35: 258-263.

24. Zhu G, Carlsen K, Carlsen KH, Lenney W, Silverman M, et al. (2008) Polymorphisms in the endothelin-1 (EDN1) are associated with asthma in two populations. Genes Immun 9: 23-29.

25. Daley D, Lemire M, Akhabir L, Chan-Yeung M, He JQ, et al. (2009) Analyses of associations with asthma in four asthma population samples from Canada and Australia. Hum Genet 125: 445-459.

26. Immervoll T, Loesgen S, Dutsch G, Gohlke H, Herbon N, et al. (2001) Fine mapping and single nucleotide polymorphism association results of candidate genes for asthma and related phenotypes. Hum Mutat 18: 327-336.

27. Kormann MS, Carr D, Klopp N, Illig T, Leupold W, et al. (2005) G-Protein-coupled receptor polymorphisms are associated with asthma in a large German population. Am J Respir Crit Care Med 171: 1358-1362.

28. Melen E, Bruce S, Doekes G, Kabesch M, Laitinen T, et al. (2005) Haplotypes of G protein-coupled receptor 154 are associated with childhood allergy and asthma. Am J Respir Crit Care Med 171: 1089-1095.

29. Malerba G, Lindgren CM, Xumerle L, Kiviluoma P, Trabetti E, et al. (2007) Chromosome 7p linkage and GPR154 gene association in Italian families with allergic asthma. Clin Exp Allergy 37: 83-89.

30. Zhu HY, Wu JM, Cui TP (2007) [Study on the association of single nucleotide polymorphisms and haplotypes of GPR154 gene with allergic asthma in Han nationality in Hubei Chinese population]. Zhonghua Yi Xue Yi Chuan Xue Za Zhi 24: 48-51.

31. Li YF, Gauderman WJ, Conti DV, Lin PC, Avol E, et al. (2008) Glutathione S-transferase P1, maternal smoking, and asthma in children: a haplotype-based analysis. Environ Health Perspect 116: 409-415.

32. Islam T, Berhane K, McConnell R, Gauderman WJ, Avol E, et al. (2009) Glutathione-S-transferase (GST) P1, GSTM1, exercise, ozone and asthma incidence in school children. Thorax 64: 197-202.

33. Fryer AA, Bianco A, Hepple M, Jones PW, Strange RC, et al. (2000) Polymorphism at the glutathione S-transferase GSTP1 locus. A new marker for bronchial hyperresponsiveness and asthma. Am J Respir Crit Care Med 161: 1437-1442.

34. Aynacioglu AS, Nacak M, Filiz A, Ekinci E, Roots I (2004) Protective role of glutathione S-transferase P1 (GSTP1) Val105Val genotype in patients with bronchial asthma. Br J Clin Pharmacol 57: 213-217.

35. Lee YL, Hsiue TR, Lee YC, Lin YC, Guo YL (2005) The association between glutathione S-transferase P1, M1 polymorphisms and asthma in Taiwanese schoolchildren. Chest 128: 1156-1162.

36. Hanene C, Jihene L, Jamel A, Kamel H, Agnes H (2007) Association of GST genes polymorphisms with asthma in Tunisian children. Mediators Inflamm 2007: 19564.

37. Kamada F, Mashimo Y, Inoue H, Shao C, Hirota T, et al. (2007) The GSTP1 gene is a susceptibility gene for childhood asthma and the GSTM1 gene is a modifier of the GSTP1 gene. Int Arch Allergy Immunol 144: 275-286.

38. Imboden M, Rochat T, Brutsche M, Schindler C, Downs SH, et al. (2008) Glutathione S-transferase genotype increases risk of progression from bronchial hyperresponsiveness to asthma in adults. Thorax 63: 322-328.

39. Randolph AG, Lange C, Silverman EK, Lazarus R, Silverman ES, et al. (2004) The IL12B gene is associated with asthma. Am J Hum Genet 75: 709-715.

40. Hirota T, Suzuki Y, Hasegawa K, Obara K, Matsuda A, et al. (2005) Functional haplotypes of IL-12B are associated with childhood atopic asthma. J Allergy Clin Immunol 116: 789-795.

41. Wjst M, Altmuller J, Faus-Kessler T, Braig C, Bahnweg M, et al. (2006) Asthma families show transmission disequilibrium of gene variants in the vitamin D metabolism and signalling pathway. Respir Res 7: 60.

42. Kim HB, Kang MJ, Lee SY, Jin HS, Kim JH, et al. (2008) Combined effect of tumour necrosis factor-alpha and interleukin-13 polymorphisms on bronchial hyperresponsiveness in Korean children with asthma. Clin Exp Allergy 38: 774-780.

43. Black S, Teixeira AS, Loh AX, Vinall L, Holloway JW, et al. (2009) Contribution of functional variation in the IL13 gene to allergy, hay fever and asthma in the NSHD longitudinal 1946 birth cohort. Allergy 64: 1172-1178.

44. Hosseini-Farahabadi S, Tavakkol-Afshari J, Rafatpanah H, Farid Hosseini R, Khaje Daluei M (2007) Association between the polymorphisms of IL-4 gene promoter (-590C>T), IL-13 coding region (R130Q) and IL-16 gene promoter (-295T>C) and allergic asthma. Iran J Allergy Asthma Immunol 6: 9-14.

45. Xi D, Pan S, Cui T, Wu J (2004) Association between IL-13 gene polymorphism and asthma in Han nationality in Hubei Chinese population. J Huazhong Univ Sci Technolog Med Sci 24: 219-222.

46. Heinzmann A, Mao XQ, Akaiwa M, Kreomer RT, Gao PS, et al. (2000) Genetic variants of IL-13 signalling and human asthma and atopy. Hum Mol Genet 9: 549-559.

47. Moissidis I, Chinoy B, Yanamandra K, Napper D, Thurmon T, et al. (2005) Association of IL-13, RANTES, and leukotriene C4 synthase gene promoter polymorphisms with asthma and/or atopy in African Americans. Genet Med 7: 406-410.

48. Howard TD, Whittaker PA, Zaiman AL, Koppelman GH, Xu J, et al. (2001) Identification and association of polymorphisms in the interleukin-13 gene with asthma and atopy in a Dutch population. Am J Respir Cell Mol Biol 25: 377-384.

49. Kamali-Sarvestani E, Ghayomi MA, Nekoee A (2007) Association of TNF-alpha -308 G/A and IL-4 -589 C/T gene promoter polymorphisms with asthma susceptibility in the south of Iran. J Investig Allergol Clin Immunol 17: 361-366.

50. Chiang CH, Tang YC, Lin MW, Chung MY (2007) Association between the IL-4 promoter polymorphisms and asthma or severity of hyperresponsiveness in Taiwanese. Respirology 12: 42-48.

51. Gervaziev YV, Kaznacheev VA, Gervazieva VB (2006) Allelic polymorphisms in the interleukin-4 promoter regions and their association with bronchial asthma among the Russian population. Int Arch Allergy Immunol 141: 257-264.

52. Schubert K, von Bonnsdorf H, Burke M, Ahlert I, Braun S, et al. (2006) A comprehensive candidate gene study on bronchial asthma and juvenile idiopathic arthritis. Dis Markers 22: 127-132.

53. Kabesch M, Tzotcheva I, Carr D, Hofler C, Weiland SK, et al. (2003) A complete screening of the IL4 gene: novel polymorphisms and their association with asthma and IgE in childhood. J Allergy Clin Immunol 112: 893-898.

54. Beghe B, Barton S, Rorke S, Peng Q, Sayers I, et al. (2003) Polymorphisms in the interleukin-4 and interleukin-4 receptor alpha chain genes confer susceptibility to asthma and atopy in a Caucasian population. Clin Exp Allergy 33: 1111-1117.

55. Noguchi E, Nukaga-Nishio Y, Jian Z, Yokouchi Y, Kamioka M, et al. (2001) Haplotypes of the 5' region of the IL-4 gene and SNPs in the intergene sequence between the IL-4 and IL-13 genes are associated with atopic asthma. Hum Immunol 62: 1251-1257.

56. Noguchi E, Shibasaki M, Arinami T, Takeda K, Yokouchi Y, et al. (1998) Association of asthma and the interleukin-4 promoter gene in Japanese. Clin Exp Allergy 28: 449-453.

57. Chouchane L, Sfar I, Bousaffara R, El Kamel A, Sfar MT, et al. (1999) A repeat polymorphism in interleukin-4 gene is highly associated with specific clinical phenotypes of asthma. Int Arch Allergy Immunol 120: 50-55.

58. Trajkov D, Mirkovska-Stojkovikj J, Arsov T, Petlichkovski A, Strezova A, et al. (2008) Association of cytokine gene polymorphisms with bronchial asthma in Macedonians. Iran J Allergy Asthma Immunol 7: 143-156.

59. Donfack J, Schneider DH, Tan Z, Kurz T, Dubchak I, et al. (2005) Variation in conserved non-coding sequences on chromosome 5q and susceptibility to asthma and atopy. Respir Res 6: 145.

60. Isidoro-Garcia M, Davila I, Laffond E, Moreno E, Lorente F, et al. (2005) Interleukin-4 (IL4) and Interleukin-4 receptor (IL4RA) polymorphisms in asthma: a case control study. Clin Mol Allergy 3: 15.

61. Adjers K, Karjalainen J, Pessi T, Eklund C, Hurme M (2005) Epistatic effect of TLR4 and IL4 genes on the risk of asthma in females. Int Arch Allergy Immunol 138: 251-256.

62. Ober C, Leavitt SA, Tsalenko A, Howard TD, Hoki DM, et al. (2000) Variation in the interleukin 4-receptor alpha gene confers susceptibility to asthma and atopy in ethnically diverse populations. Am J Hum Genet 66: 517-526.

63. Zhang W, Zhang X, Qiu D, Sandford A, Tan WC (2007) IL-4 receptor genetic polymorphisms and asthma in Asian populations. Respir Med 101: 186-190.

64. Zhang H, Zhang Q, Wang L, Chen H, Li Y, et al. (2007) Association of IL4R gene polymorphisms with asthma in Chinese populations. Hum Mutat 28: 1046.

65. Hytonen AM, Lowhagen O, Arvidsson M, Balder B, Bjork AL, et al. (2004) Haplotypes of the interleukin-4 receptor alpha chain gene associate with susceptibility to and severity of atopic asthma. Clin Exp Allergy 34: 1570-1575.

66. Zhang AM, Li HL, Hao P, Chen YH, Li JH, et al. (2006) [Association of Q576R polymorphism in the interleukin-4 receptor gene with serum IgE levels in children with asthma]. Zhongguo Dang Dai Er Ke Za Zhi 8: 109-112.

67. Zhu G, Vestbo J, Lenney W, Silverman M, Whyte M, et al. (2007) Association of PTGDR gene polymorphisms with asthma in two Caucasian populations. Genes Immun 8: 398-403.

68. Sanz C, Isidoro-Garcia M, Davila I, Moreno E, Laffond E, et al. (2006) Promoter genetic variants of prostanoid DP receptor (PTGDR) gene in patients with asthma. Allergy 61: 543-548.

69. Oguma T, Palmer LJ, Birben E, Sonna LA, Asano K, et al. (2004) Role of prostanoid DP receptor variants in susceptibility to asthma. N Engl J Med 351: 1752-1763.

70. Li YF, Gauderman WJ, Avol E, Dubeau L, Gilliland FD (2006) Associations of tumor necrosis factor G-308A with childhood asthma and wheezing. Am J Respir Crit Care Med 173: 970-976.

71. Jimenez-Morales S, Velazquez-Cruz R, Ramirez-Bello J, Bonilla-Gonzalez E, Romero-Hidalgo S, et al. (2009) Tumor necrosis factor-alpha is a common genetic risk factor for asthma, juvenile rheumatoid arthritis, and systemic lupus erythematosus in a Mexican pediatric population. Hum Immunol 70: 251-256.

72. Sharma S, Sharma A, Kumar S, Sharma SK, Ghosh B (2006) Association of TNF haplotypes with asthma, serum IgE levels, and correlation with serum TNF-alpha levels. Am J Respir Cell Mol Biol 35: 488-495.

73. Migita O, Noguchi E, Koga M, Jian Z, Shibasaki M, et al. (2005) Haplotype analysis of a 100 kb region spanning TNF-LTA identifies a polymorphism in the LTA promoter region that is associated with atopic asthma susceptibility in Japan. Clin Exp Allergy 35: 790-796.

74. Wang TN, Chen WY, Wang TH, Chen CJ, Huang LY, et al. (2004) Gene-gene synergistic effect on atopic asthma: tumour necrosis factor-alpha-308 and lymphotoxin-alpha-NcoI in Taiwan's children. Clin Exp Allergy 34: 184-188.

75. Noguchi E, Yokouchi Y, Shibasaki M, Inudou M, Nakahara S, et al. (2002) Association between TNFA polymorphism and the development of asthma in the Japanese population. Am J Respir Crit Care Med 166: 43-46.

76. Moffatt MF, James A, Ryan G, Musk AW, Cookson WO (1999) Extended tumour necrosis factor/HLA-DR haplotypes and asthma in an Australian population sample. Thorax 54: 757-761.

77. Moffatt MF, Cookson WO (1997) Tumour necrosis factor haplotypes and asthma. Hum Mol Genet 6: 551-554.

78. Wu H, Romieu I, Sienra-Monge JJ, del Rio-Navarro BE, Anderson DM, et al. (2007) Parental smoking modifies the relation between genetic variation in tumor necrosis factor-alpha (TNF) and childhood asthma. Environ Health Perspect 115: 616-622.

79. Albuquerque RV, Hayden CM, Palmer LJ, Laing IA, Rye PJ, et al. (1998) Association of polymorphisms within the tumour necrosis factor (TNF) genes and childhood asthma. Clin Exp Allergy 28: 578-584.

80. Randolph AG, Lange C, Silverman EK, Lazarus R, Weiss ST (2005) Extended haplotype in the tumor necrosis factor gene cluster is associated with asthma and asthma-related phenotypes. Am J Respir Crit Care Med 172: 687-692.

81. Witte JS, Palmer LJ, O'Connor RD, Hopkins PJ, Hall JM (2002) Relation between tumour necrosis factor polymorphism TNFalpha-308 and risk of asthma. Eur J Hum Genet 10: 82-85.

82. Shin HD, Park BL, Kim LH, Jung JH, Wang HJ, et al. (2004) Association of tumor necrosis factor polymorphisms with asthma and serum total IgE. Hum Mol Genet 13: 397-403.

83. Winchester EC, Millwood IY, Rand L, Penny MA, Kessling AM (2000) Association of the TNF-alpha-308 (G-->A) polymorphism with self-reported history of childhood asthma. Hum Genet 107: 591-596.

84. Gupta V, Sarin BC, Changotra H, Sehajpal PK (2005) Association of G-308A TNF-alpha polymorphism with bronchial asthma in a North Indian population. J Asthma 42: 839-841.

85. Kumar V, Khosla R, Gupta V, Sarin BC, Sehajpal PK (2008) Differential association of tumour necrosis factor-alpha single nucleotide polymorphism (-308) with tuberculosis and bronchial asthma. Natl Med J India 21: 120-122.

86. Kumar A, Gupta V, Changotra H, Sarin BC, Sehajpal PK (2008) Tumor necrosis factor--alpha and transforming growth factor--beta1 polymorphisms in bronchial asthma. Indian J Med Sci 62: 323-330.

87. Castro-Giner F, Kogevinas M, Imboden M, de Cid R, Jarvis D, et al. (2009) Joint effect of obesity and TNFA variability on asthma: two international cohort studies. Eur Respir J 33: 1003-1009.

88. Castro-Giner F, Kogevinas M, Machler M, de Cid R, Van Steen K, et al. (2008) TNFA -308G>A in two international population-based cohorts and risk of asthma. Eur Respir J 32: 350-361.

89. Munthe-Kaas MC, Carlsen KL, Carlsen KH, Egeland T, Haland G, et al. (2007) HLA Dr-Dq haplotypes and the TNFA-308 polymorphism: associations with asthma and allergy. Allergy 62: 991-998.

90. Raby BA, Lazarus R, Silverman EK, Lake S, Lange C, et al. (2004) Association of vitamin D receptor gene polymorphisms with childhood and adult asthma. Am J Respir Crit Care Med 170: 1057-1065.

91. Saadi A, Gao G, Li H, Wei C, Gong Y, et al. (2009) Association study between vitamin D receptor gene polymorphisms and asthma in the Chinese Han population: a case-control study. BMC Med Genet 10: 71.

92. Poon AH, Laprise C, Lemire M, Montpetit A, Sinnett D, et al. (2004) Association of vitamin D receptor genetic variants with susceptibility to asthma and atopy. Am J Respir Crit Care Med 170: 967-973.
