# Supplementary material for: Unifying Candidate Gene and GWAS Approaches in Asthma
Source: PLoS One. 2010 Nov 12;5(11):e13894. doi: 10.1371/journal.pone.0013894 (PMC2980484; doi:10.1371/journal.pone.0013894)
Supplement: Table S2 — Candidate gene associations with asthma and comparison between genotyping and imputation results. Odds ratios, 95% confidence intervals are given for SNP effects. R2 is given for SNPs were both imputation and genotyping data is available. Bold font indicates previously reported SNPs, standard font is tagging SNPs, cursive font indicates SNPs that are neither tagging nor previously reported SNPs; * p<0.05; **p<0.0035 (0.10 MB PDF) [file pone.0013894.s002.pdf]

ADAM33	SNPs	minor allele	OR genotyping	OR imputation HM2 (HM3)	CI_L 95% genotyping	CI_L 95% imputation HM2 (HM3)	CI_U 95% genotyping	CI_U 95% imputation HM2 (HM3)	p-value genotyping	p-value imputation HM2 (HM3)	R2
	rs6084432	A	1.177	1.177 (1.177)	0.965	0.965 (0.9648)	1.436	1.436 (1.436)	0.108	0.108 (0.1082)	1
	rs12624874	T		1.093 (1.026)		0.871 (0.8133)		1.372 (1.295)		0.441 (0.827)	
	rs633330	T		2.12		0.65		6.92		0.21	
	rs630909	A		1.11		0.91		1.35		0.33	
	rs512625	A	1.037	1.046 (1.037)	0.886	0.891 (0.8856)	1.215	1.229 (1.215)	0.649	0.584 (0.6492)	0.95
	rs2787093	C		0.98		0.78		1.23		0.86	
	**rs3746631**	**C**	**1.040**	**1.034 (1.011)**	**0.669**	**0.619 (0.5807)**	**1.616**	**1.730 (1.761)**	**0.862**	**0.898 (0.9683)**	**0.31**
	**rs2787094**	**C**	**1.122**		**0.937**		**1.342**		**0.211**		
	rs677044	G		0.9		0.74		1.1		0.31	
	**rs3918400**	**A**	**0.975**	**1.03**	**0.645**	**0.62**	**1.473**	**1.73**	**0.904**	**0.9**	**0.30**
	**rs543749**	**T**	**1.200**		**0.964**		**1.493**		**0.103**		
	**rs628977**	-	-	-	-	-	-	-	-	-	-
	rs678881	C		0.91		0.76		1.09		0.29	
	**rs2280089**	**A**	**1.161**	**1.160 (1.18)**	**0.928**	**0.934 (0.9475)**	**1.452**	**1.441 (1.469)**	**0.191**	**0.180 (0.1395)**	**0.86**
	**rs2280090**	**A**	**1.105**		**0.894**		**1.366**		**0.357**		
	**rs2280091**	**A**		**1.093 (1.126)**		**0.886 (0.9067)**		**1.350 (1.399)**		**0.407 (0.2823)**	
	**rs574174**	**T**	**1.149**		**0.957**		**1.379**		**0.138**		
	**rs44707**	**G**	**0.895**		**0.763**		**1.050**		**0.173**		
	**rs598418**	**G**		**0.924 (0.99)**		**0.787 (0.8469)**		**1.084 (1.158)**		**0.330 (0.9007)**	
	**rs2853209**	**A**	**1.042**		**0.887**		**1.224**		**0.619**		
	**rs528557 ****	**G**	**1.504**		**1.247**		**1.813**		**0.000019**		
	**rs3918396**	**T**	**1.291**		**0.996**		**1.672**		**0.054**		
	**rs612709**	**A**	**1.177**		**0.948**		**1.461**		**0.139**		
	**rs3918395**	**A**	**1.123**	**1.059 (1.118)**	**0.894**	**0.856 (0.8969)**	**1.411**	**1.310 (1.394)**	**0.320**	**0.600 (0.3211)**	**0.67**
	**rs2280092**	-	-	-	-	-	-	-	-	-	-
	**rs511898**	**T**	**1.127**	**(1.103)**	**0.961**	**(0.9399)**	**1.323**	**(1.294)**	**0.142**	**(0.23)**	
	rs2787095	C		1.07		0.91		1.26		0.4	
	rs621394	A		2.12		0.65		6.92		0.21	
	rs2853210	T		0.939 (0.9078)		0.773 (0.7463)		1.140 (1.104)		0.523 (0.3336)	
	rs4987246	G		1.68		0.74		3.83		0.22	
	rs487377	A	1.054	1.071 (1.054)	0.879	0.894 (0.8794)	1.264	1.284 (1.264)	0.569	0.455 (0.5679)	0.98
	rs570269	C		0.99		0.81		1.22		0.94	
	rs7354032	T		0.774 (1.029)		0.507 (0.8771)		1.184 (1.208)		0.238 (0.7237)	
	rs554743	G	1.038	1.03	0.881	0.88	1.222	1.21	0.658	0.71	0.98
	rs2853215	A	1.085	1.085 (1.082)	0.917	0.917 (0.9141)	1.284	1.284 (1.28)	0.340	0.339 (0.3607)	1
	rs8124504	T		1.01		0.64		1.6		0.96	
	rs532448	T		1		0.86		1.17		0.97	
	rs8124665	G		1.3		0.68		2.5		0.43	

CCL5	SNPs	minor allele	OR genotyping	OR imputation HM2 (HM3)	CI_L 95% genotyping	CI_L 95% imputation HM2 (HM3)	CI_U 95% genotyping	CI_U 95% imputation HM2 (HM3)	p-value genotyping	p-value imputation HM2 (HM3)	R2
	rs16963927	G		0.649 (0.8788)		0.380 (0.6258)		1.106 (1.234)		0.112 (0.4554)	
	**rs2280788**	**C**	**1.217**	**0.75**	**0.775**	**0.2**	**1.912**	**2.8**	**0.393**	**0.67**	**0.12**
	rs1800825	G		1.12		0.34		3.7		0.85	
	**rs2107538**	**T**	**1.190**	**1.088 (1.095)**	**0.978**	**0.889 (0.9086)**	**1.448**	**1.331 (1.32)**	**0.082**	**0.412 (0.3395)**	**0.78**
	rs4239253	C		1.22		0.53		2.8		0.64	
	rs4796123	C		1.204 (1.175)		0.970 (0.954)		1.495 (1.448)		0.093 (0.1291)	

CD14	SNPs	minor allele	OR genotyping	OR imputation HM2 (HM3)	CI_L 95% genotyping	CI_L 95% imputation HM2 (HM3)	CI_U 95% genotyping	CI_U 95% imputation HM2 (HM3)	p-value genotyping	p-value imputation HM2 (HM3)	R2
	rs778588	C		0.970 (0.9388)		0.826 (0.7982)		1.139 (1.104)		0.711 (0.446)	
	rs11574651	A		0.47		0.12		1.87		0.28	
	**rs4914**	**G**		**0.92**		**0.75**		**1.13**		**0.43**	
	**rs2569190**	**A**	**1.011**	**0.99**	**0.865**	**0.85**	**1.182**	**1.16**	**0.890**	**0.92**	**0.83**
	rs5744448	T		2.196 (0.6662)		0.566 (0.2104)		8.527 (2.109)		0.256 (0.4898)	
	rs5744442	C		0.86		0.6		1.23		0.4	
	rs5744441	A		1.031 (1.06)		0.864 (0.8885)		1.231 (1.264)		0.734 (0.5183)	

DPP10	SNPs	minor allele	OR genotyping	OR imputation HM2 (HM3)	CI_L 95% genotyping	CI_L 95% imputation HM2 (HM3)	CI_U 95% genotyping	CI_U 95% imputation HM2 (HM3)	p-value genotyping	p-value imputation HM2 (HM3)	R2
	**rs1036022 (rs13392783 / r²=0.88)**	**C**	**0.939**	**0.94**	**0.808**	**0.81**	**1.092**	**1.1**	**0.416**	**0.45**	**1**
	rs10208402	C		0.944 (0.9554)		0.814 (0.8242)		1.095 (1.107)		0.445 (0.5443)	
	rs1367178	G		1.005 (0.9918)		0.654 (0.669)		1.542 (1.47)		0.984 (0.9675)	
	rs17048357	T		1.1		0.57		2.13		0.77	
	rs17048359	C		0.976 (1.008)		0.823 (0.8546)		1.157 (1.189)		0.778 (0.9244)	
	rs10192393	C	1.053	1.057 (1.053)	0.906	0.909 (0.9059)	1.224	1.229 (1.224)	0.500	0.475 (0.5003)	1
	rs1430092	A		0.85		0.6		1.2		0.36	
	rs12473953	C		1.005 (0.9894)		0.857 (0.8428)		1.180 (1.161)		0.948 (0.8963)	
	rs1430091	A		1.04		0.86		1.28		0.67	
	rs4849333	C		0.937 (0.8835)		0.776 (0.7329)		1.131 (1.065)		0.499 (0.1937)	
	rs1430090	*G*	*1.049*	1.040 (1.032)	*0.891*	0.884 (0.8776)	*1.236*	1.224 (1.214)	*0.565*	0.638 (0.7014)	*1*
	rs6737251	*T*	*0.975*	0.975 (0.9753)	*0.833*	0.833 (0.833)	*1.142*	1.142 (1.142)	*0.756*	0.756 (0.7557)	*1*
	rs17048372	T		1.02		0.84		1.23		0.88	
	rs6709636	A		1.41		0.4		5.01		0.6	
	rs11685217	*T*	*1.006*	1.006 (1.006)	*0.833*	0.833 (0.8327)	*1.216*	1.216 (1.216)	*0.947*	0.947 (0.9469)	*1*
	rs1430089	G		0.98		0.84		1.15		0.78	
	rs17764091	G		1.006 (0.9758)		0.802 (0.7821)		1.261 (1.218)		0.962 (0.8284)	
	rs17764121 *	C		5.21		1.15		23.61		0.03	
	rs17048408	C		0.73		0.37		1.46		0.38	
	**rs7559909 (rs13011555 / r²=0.9)**	**A**	**0.946**	**0.944 (0.9443)**	**0.811**	**0.809 (0.8092)**	**1.104**	**1.102 (1.102)**	**0.483**	**0.467 (0.4667)**	**1**
	rs17048446	A		0.65		0.4		1.06		0.08	
	rs17048451	C		0.56		0.2		1.54		0.26	
	rs10496465	G	0.929	0.931 (0.9307)	0.755	0.757 (0.7565)	1.143	1.145 (1.145)	0.486	0.497 (0.4968)	1
	rs4849338	C		1		0.86		1.18		0.96	
	rs13397905	C		0.56		0.13		2.35		0.43	
	rs1820924	T		1.032 (0.9804)		0.849 (0.8099)		1.255 (1.187)		0.749 (0.8393)	
	rs6750402	C	1.006	1.012 (1.008)	0.847	0.852 (0.8486)	1.195	1.202 (1.197)	0.946	0.892 (0.9285)	1
	rs10187644	T		0.920 (0.9084)		0.792 (0.7813)		1.070 (1.056)		0.278 (0.2117)	
	rs1346762	G	1.110	1.112 (1.112)	0.947	0.950 (0.9495)	1.300	1.303 (1.303)	0.197	0.188 (0.1877)	1
	rs1346763	T		1.096 (1.056)		0.940 (0.9059)		1.278 (1.231)		0.241 (0.4862)	
	rs1430101	*G*	*1.043*	1.028 (1.03)	*0.866*	0.853 (0.8559)	*1.256*	1.239 (1.24)	*0.661*	0.772 (0.7535)	*0.97*
	rs17048470	A		0.68		0.37		1.23		0.2	
	rs2420165	T		0.993 (0.8931)		0.743 (0.6958)		1.325 (1.146)		0.960 (0.3744)	
	rs11674031	G		0.857 (0.8823)		0.709 (0.7259)		1.036 (1.072)		0.110 (0.2084)	
	rs1430103	G		1.042 (1.034)		0.859 (0.8594)		1.264 (1.245)		0.675 (0.7215)	
	rs6746254	T		1.089 (1.073)		0.863 (0.8618)		1.374 (1.335)		0.471 (0.5304)	
	rs10048644	A		0.97		0.75		1.27		0.84	
	rs11123246	T		0.944 (0.8862)		0.742 (0.7287)		1.201 (1.078)		0.638 (0.2263)	
	rs1430108	C	1.006	1.009 (1.003)	0.865	0.867 (0.8621)	1.170	1.173 (1.166)	0.940	0.912 (0.9733)	1
	rs6542214	C	0.983	0.985 (0.9856)	0.844	0.845 (0.8459)	1.146	1.148 (1.148)	0.828	0.842 (0.8521)	0.97
	rs12987334	C		0.865 (0.9043)		0.652 (0.6871)		1.147 (1.19)		0.314 (0.4731)	
	rs1835328	T		0.99		0.83		1.18		0.93	
	rs7590021	C		0.972 (1.033)		0.790 (0.8503)		1.196 (1.255)		0.786 (0.7436)	
	rs6741327	A		1.08		0.88		1.31		0.47	
	rs10514812	A	1.219	1.214 (1.221)	0.947	0.942 (0.9488)	1.569	1.564 (1.572)	0.124	0.134 (0.1207)	1
	rs10496466	A	1.007	1.002 (1.015)	0.829	0.822 (0.8359)	1.224	1.221 (1.233)	0.941	0.985 (0.879)	0.97
	rs6729737	G	1.031	1.035 (1.031)	0.880	0.883 (0.8801)	1.209	1.213 (1.208)	0.705	0.672 (0.7034)	1
	rs996135	A	1.090	1.094 (1.093)	0.917	0.921 (0.9205)	1.295	1.299 (1.299)	0.328	0.307 (0.3091)	1
	rs12999482	A		0.855 (0.8485)		0.667 (0.6613)		1.098 (1.089)		0.220 (0.1965)	
	rs1965088	G	1.002	1.000 (1.005)	0.827	0.825 (0.8291)	1.213	1.211 (1.218)	0.987	0.998 (0.9617)	1
	rs17715313	C		0.98		0.68		1.43		0.93	
	rs7582739	C	1.217	1.167 (1.169)	0.939	0.904 (0.905)	1.576	1.506 (1.509)	0.138	0.235 (0.232)	0.99
	rs1347502	C		0.974 (0.9522)		0.808 (0.7985)		1.174 (1.136)		0.781 (0.5858)	
	rs17782840	A		1.053 (0.9971)		0.815 (0.7757)		1.360 (1.282)		0.694 (0.9818)	
	rs785033	C		1.02		0.87		1.2		0.81	
	rs785041	G		1.227 (1.093)		0.885 (0.8045)		1.702 (1.484)		0.220 (0.5708)	
	rs17715765	G		0.98		0.63		1.53		0.94	
	rs13029707	T		0.94		0.4		2.17		0.88	
	rs17715867	C		1.048 (1.057)		0.732 (0.7603)		1.502 (1.47)		0.797 (0.7406)	
	rs6542217	A	0.982	0.985 (0.9781)	0.832	0.835 (0.8289)	1.159	1.163 (1.154)	0.829	0.861 (0.7931)	1
	rs17715987	T		1		0.66		1.51		0.98	
	rs12711800	C	0.989	0.982 (0.9906)	0.822	0.816 (0.8233)	1.190	1.183 (1.192)	0.906	0.850 (0.9203)	0.99
	rs12996109	T	0.947	0.95	0.761	0.76	1.179	1.18	0.629	0.63	1
	rs6542218	T		1.069 (0.9652)		0.760 (0.6735)		1.503 (1.383)		0.702 (0.8471)	
	rs13030268	A		1.23		0.76		2		0.41	
	rs7575903	T		0.933 (0.9401)		0.737 (0.7357)		1.181 (1.201)		0.564 (0.6215)	
	rs13425741	A		1.01		0.83		1.23		0.91	
	rs10496470	A	0.964	0.977 (0.9768)	0.747	0.758 (0.7575)	1.244	1.259 (1.259)	0.777	0.856 (0.8562)	1
	rs9308697	*C*	*0.984*	0.989 (0.9748)	*0.818*	0.822 (0.8104)	*1.183*	1.191 (1.173)	*0.862*	0.910 (0.7865)	*0.98*
	rs17783638	C		0.980 (0.918)		0.714 (0.6568)		1.345 (1.283)		0.900 (0.6167)	
	rs11695555	G		0.79		0.41		1.52		0.48	
	rs13022574	*A*	*0.923*	0.923 (0.9227)	*0.691*	0.691 (0.6909)	*1.232*	1.232 (1.232)	*0.586*	0.586 (0.5859)	*1*
	rs1013261	G		0.99		0.77		1.27		0.92	
	rs1561070	A	1.029	0.960 (0.9502)	0.797	0.750 (0.727)	1.327	1.228 (1.242)	0.829	0.744 (0.7085)	1
	rs17783822	G		0.98		0.72		1.34		0.89	
	rs10175018	G	0.941	0.947 (0.9466)	0.776	0.780 (0.7804)	1.140	1.151 (1.148)	0.533	0.586 (0.5777)	0.97
	rs718062	T	1.070	1.079 (1.079)	0.912	0.921 (0.9205)	1.255	1.266 (1.266)	0.407	0.347 (0.347)	1
	rs17043340	G		0.99		0.67		1.44		0.94	
	rs7589409	G	1.070	1.070 (1.074)	0.921	0.921 (0.9237)	1.244	1.244 (1.249)	0.376	0.376 (0.3532)	1
	rs13430153	C	1.024	1.024 (1.024)	0.803	0.803 (0.8028)	1.307	1.307 (1.307)	0.847	0.847 (0.8466)	1
	rs17043361	A	0.931	0.931 (0.9305)	0.675	0.675 (0.6747)	1.283	1.283 (1.283)	0.660	0.660 (0.6604)	1
	rs930314	C	1.235	1.295 (1.278)	0.821	0.868 (0.852)	1.858	1.933 (1.918)	0.311	0.206 (0.2354)	1
	rs10190695	G	1.115	1.115 (1.112)	0.959	0.959 (0.9558)	1.298	1.297 (1.293)	0.158	0.158 (0.1699)	1
	rs4569486	T		1.29		0.82		2.03		0.28	
	rs7594265	G		1.12		0.96		1.3		0.16	
	rs1435878	T	1.085	1.081 (1.084)	0.928	0.925 (0.928)	1.267	1.263 (1.267)	0.307	0.327 (0.3079)	1
	rs17784852	C		0.875 (1.012)		0.640 (0.7459)		1.195 (1.374)		0.400 (0.9369)	
	rs13030450	G	0.815	0.815 (0.8145)	0.580	0.580 (0.58)	1.144	1.144 (1.144)	0.236	0.236 (0.2361)	1
	rs17728568	G	1.002	0.984 (1.001)	0.689	0.676 (0.6901)	1.457	1.433 (1.451)	0.993	0.934 (0.9976)	0.99
	rs7606549	*A*	*1.131*	1.131 (1.131)	*0.973*	0.973 (0.9725)	*1.315*	1.315 (1.315)	*0.109*	0.110 (0.1102)	*1*
	rs17043456	G		1.165 (1.153)		0.897 (0.8889)		1.514 (1.495)		0.252 (0.2837)	
	rs1435879	G	0.923	0.941 (0.9409)	0.734	0.749 (0.7489)	1.162	1.182 (1.182)	0.495	0.601 (0.6011)	1
	rs7574829	*G*	*1.125*	1.125 (1.125)	*0.851*	0.851 (0.8507)	*1.487*	1.487 (1.487)	*0.409*	0.409 (0.4092)	*1*
	rs2117851	T	0.985	0.984 (1.01)	0.832	0.831 (0.8544)	1.167	1.164 (1.193)	0.864	0.847 (0.9099)	1
	rs7607329	A		0.41		0.13		1.35		0.14	
	rs2082875	A	1.111	1.09	0.952	0.94	1.297	1.27	0.180	0.26	1
	rs17729194	A		0.73		0.33		1.62		0.44	
	rs10211528	G		1.066 (1.113)		0.793 (0.829)		1.432 (1.495)		0.674 (0.4752)	
	rs4849350	A		0.95		0.74		1.21		0.65	
	rs1435868	T	1.036	1.039 (1.03)	0.895	0.899 (0.8905)	1.199	1.202 (1.192)	0.638	0.601 (0.6901)	0.97
	rs10187047	G		1.2		0.82		1.78		0.35	
	rs11890570	C		1.105 (1.198)		0.628 (0.7335)		1.947 (1.957)		0.728 (0.4702)	
	rs319853	G	0.941	0.943 (0.9434)	0.812	0.814 (0.8143)	1.090	1.093 (1.093)	0.415	0.438 (0.4378)	1
	rs319854	G	0.941	0.943 (0.9432)	0.812	0.814 (0.8142)	1.090	1.093 (1.093)	0.414	0.436 (0.4364)	1
	rs17786162	C		1.070 (1.065)		0.783 (0.7715)		1.464 (1.469)		0.670 (0.7032)	
	rs319858	C		1.045 (1.046)		0.902 (0.9033)		1.210 (1.211)		0.557 (0.5491)	
	rs368872	G	0.934	0.937 (0.9359)	0.807	0.810 (0.8083)	1.082	1.085 (1.084)	0.363	0.384 (0.376)	0.99
	rs10496474	A		1.08		0.79		1.46		0.64	
	rs6754133	C	1.080	1.092 (1.092)	0.810	0.818 (0.8184)	1.440	1.457 (1.457)	0.602	0.549 (0.5492)	0.99
	rs17043547	A		1.179 (1.192)		0.606 (0.6468)		2.296 (2.197)		0.628 (0.5732)	
	rs319851	G	1.008	1.007 (1.007)	0.869	0.868 (0.8684)	1.169	1.168 (1.168)	0.917	0.924 (0.9242)	1
	rs12478225	G		1.22		0.87		1.71		0.26	
	rs12711805	T		1.04		0.89		1.21		0.61	
	rs7582954	A	1.048	1.045 (1.039)	0.905	0.902 (0.897)	1.214	1.211 (1.203)	0.531	0.557 (0.6111)	1
	rs6726425	*T*	*1.049*	1.045 (1.042)	*0.905*	0.902 (0.8995)	*1.215*	1.211 (1.207)	*0.527*	0.557 (0.5839)	*1*
	rs1519668	*C*	*1.020*	1.026 (1.029)	*0.876*	0.881 (0.884)	*1.187*	1.195 (1.199)	*0.801*	0.740 (0.7095)	*1*
	rs12473375	A		1.08		0.79		1.47		0.64	
	rs10496475	A	1.141	1.129 (1.129)	0.923	0.913 (0.9125)	1.411	1.396 (1.396)	0.222	0.264 (0.2644)	0.99
	rs2420449	A	0.970	0.967 (0.97)	0.831	0.828 (0.8308)	1.132	1.129 (1.132)	0.700	0.672 (0.6996)	1
	rs908931	T		0.974 (0.982)		0.840 (0.8467)		1.130 (1.139)		0.732 (0.8097)	
	rs1519673	C	1.056	1.062 (1.059)	0.909	0.914 (0.9119)	1.226	1.233 (1.229)	0.475	0.432 (0.4533)	0.99
	rs1519674	C		1.038 (1.033)		0.872 (0.8708)		1.234 (1.226)		0.678 (0.7078)	
	rs9308702	T		1.2		0.94		1.54		0.14	
	rs1474170	T	0.932	0.928 (0.9272)	0.767	0.764 (0.7634)	1.133	1.127 (1.126)	0.481	0.449 (0.4461)	0.97
	rs13385730	T		1.88		0.47		7.55		0.37	
	rs6732028	G		0.972 (0.9694)		0.831 (0.8265)		1.137 (1.137)		0.721 (0.7025)	
	rs13421246	C		1.03		0.86		1.25		0.72	
	rs7597658	G		0.95		0.81		1.12		0.54	
	rs2139411	T	1.065	1.07	0.875	0.89	1.295	1.29	0.531	0.47	1
	rs7558870	G	0.985	0.997 (0.9756)	0.842	0.852 (0.8339)	1.151	1.166 (1.141)	0.846	0.968 (0.7578)	0.96
	rs10496476	T	1.064	1.067 (1.067)	0.893	0.896 (0.8958)	1.268	1.271 (1.271)	0.491	0.467 (0.4673)	1
	rs6755195	T	0.841	0.840 (0.8399)	0.667	0.666 (0.6658)	1.061	1.060 (1.06)	0.145	0.141 (0.141)	1
	rs13401032	A		1.32		0.8		2.17		0.27	
	rs17788273	G		0.91		0.78		1.06		0.23	
	rs1519667	G	0.923	0.923 (0.9206)	0.791	0.792 (0.7894)	1.076	1.077 (1.074)	0.306	0.310 (0.2914)	1
	rs7604124	G		1.020 (0.9982)		0.860 (0.8456)		1.208 (1.178)		0.824 (0.9834)	
	rs1879124	G		1.122 (1.13)		0.946 (0.9532)		1.330 (1.34)		0.187 (0.1592)	
	rs13020576	A	0.971	0.973 (0.9727)	0.822	0.823 (0.8233)	1.147	1.149 (1.149)	0.731	0.745 (0.7447)	1
	rs17732131	T		0.94		0.8		1.09		0.41	
	rs11684641	A		1.150 (1.19)		0.951 (0.9835)		1.391 (1.441)		0.150 (0.07362)	
	rs13007523	A	0.906	0.911 (0.9081)	0.776	0.781 (0.7787)	1.056	1.062 (1.059)	0.207	0.235 (0.2193)	1
	rs17043702	C		1.21		0.76		1.95		0.43	
	rs12471830	G		1.034 (1.043)		0.882 (0.8913)		1.213 (1.22)		0.679 (0.601)	
	rs7607352	T		0.95		0.81		1.13		0.58	
	rs998429	C	1.153	1.133 (1.133)	0.966	0.952 (0.9516)	1.375	1.350 (1.35)	0.115	0.161 (0.1605)	1
	rs17789111	G		1.1		0.79		1.52		0.58	
	rs1879123	A		0.93		0.8		1.08		0.35	
	rs17043738	G		0.62		0.18		2.21		0.46	
	rs13032069	A		1.070 (1.074)		0.889 (0.8969)		1.288 (1.285)		0.476 (0.4387)	
	rs17452256	C		1.21		0.81		1.82		0.36	
	rs970882	A		0.9		0.77		1.05		0.16	
	rs7559740	T		1.093 (0.9629)		0.845 (0.7559)		1.412 (1.227)		0.499 (0.7597)	
	rs7369383	*T*	*0.933*	0.93	*0.762*	0.76	*1.142*	1.14	*0.501*	0.5	*1*
	rs1914973	T		1.149 (1.219)		0.917 (0.9703)		1.438 (1.531)		0.227 (0.08901)	
	rs11123261	C		0.94		0.79		1.12		0.48	
	rs1914974	T		0.76		0.56		1.03		0.08	
	rs10496481	T	1.143	1.14	0.963	0.96	1.357	1.36	0.127	0.13	1
	rs1401757	T		1.05		0.9		1.23		0.52	
	rs980316	C	0.873	0.873 (0.873)	0.745	0.745 (0.7451)	1.023	1.023 (1.023)	0.092	0.092 (0.0922)	1
	rs980317	*C*	*0.949*	0.951 (0.9474)	*0.801*	0.803 (0.8005)	*1.124*	1.126 (1.121)	*0.545*	0.558 (0.5296)	*1*
	rs2175176	T	1.076	1.082 (1.082)	0.925	0.931 (0.9309)	1.251	1.258 (1.258)	0.344	0.305 (0.3039)	1
	rs2420589	G	0.865	0.87	0.738	0.74	1.014	1.01	0.075	0.08	1
	rs17043845	G		1.14		0.97		1.35		0.11	
	rs17043859	C		0.744 (0.865)		0.553 (0.7376)		1.001 (1.014)		0.051 (0.075)	
	rs6745631	C	0.937	0.943 (0.943)	0.791	0.796 (0.7961)	1.110	1.117 (1.117)	0.454	0.496 (0.4961)	1
	rs2089674	G		0.86		0.59		1.27		0.46	
	rs1509739	G	1.012	1.037 (1.037)	0.836	0.861 (0.8612)	1.225	1.249 (1.249)	0.904	0.700 (0.7003)	1
	rs2036779	T	1.171	1.168 (1.183)	0.944	0.942 (0.9537)	1.452	1.448 (1.467)	0.151	0.157 (0.127)	1
	rs1396932	*A*	*0.935*	0.935 (0.933)	*0.804*	0.804 (0.802)	*1.088*	1.088 (1.085)	*0.385*	0.387 (0.367)	*1*
	rs1396933	A		0.94		0.8		1.09		0.39	
	rs949577	*C*	*0.899*	0.901 (0.8973)	*0.749*	0.752 (0.7483)	*1.078*	1.081 (1.076)	*0.248*	0.262 (0.2422)	*1*
	rs7603116	G		0.972 (0.9434)		0.781 (0.7562)		1.209 (1.177)		0.796 (0.6061)	
	rs1396934	A	0.945	0.945 (0.9446)	0.776	0.776 (0.7762)	1.149	1.149 (1.149)	0.569	0.569 (0.5691)	1
	rs17355229	G		0.95		0.66		1.37		0.79	
	rs17452458	G		1.095 (1.109)		0.915 (0.9299)		1.311 (1.323)		0.322 (0.2496)	
	rs17452465	T		1.41		0.84		2.36		0.2	
	rs6542233	A		0.88		0.73		1.06		0.17	
	rs7570895	T		0.96		0.8		1.15		0.64	
	rs1980007	A	0.970	0.973 (0.9718)	0.814	0.816 (0.8151)	1.156	1.160 (1.159)	0.733	0.760 (0.75)	0.98
	rs1876860	G		1.038 (1.067)		0.880 (0.9072)		1.224 (1.255)		0.662 (0.4328)	
	rs13411309	T		0.947 (1.013)		0.720 (0.7893)		1.245 (1.301)		0.695 (0.9166)	
	rs1396921	G		1.14		0.97		1.35		0.11	
	rs6720401	T		0.944 (0.8556)		0.739 (0.6639)		1.207 (1.103)		0.647 (0.2281)	
	rs12611989	A		1.010 (0.9885)		0.872 (0.8535)		1.170 (1.145)		0.893 (0.8772)	
	rs12618420	T		0.8		0.51		1.25		0.32	
	rs12998470	G		1.31		0.86		2		0.21	
	rs1396936 *	A	0.702	0.711 (0.7112)	0.511	0.518 (0.5183)	0.965	0.976 (0.9759)	0.029	0.035 (0.03476)	1
	rs12328529	G		0.801 (0.9143)		0.534 (0.6262)		1.203 (1.335)		0.286 (0.6428)	
	rs11694256	*T*	*1.033*	1.030 (1.027)	*0.885*	0.883 (0.8804)	*1.205*	1.201 (1.197)	*0.684*	0.707 (0.7372)	*1*
	rs1396925	A		0.93		0.8		1.08		0.36	
	rs13415710	G		0.72		0.46		1.1		0.13	
	rs1396930	A		1.16		0.69		1.97		0.58	
	rs6709267	*A*	*1.038*	1.035 (1.035)	*0.891*	0.889 (0.889)	*1.209*	1.206 (1.206)	*0.632*	0.655 (0.6548)	*1*
	rs17452616	T		0.987 (0.9866)		0.851 (0.8506)		1.144 (1.144)		0.858 (0.8583)	
	rs2901277	C		0.891 (0.9229)		0.728 (0.7534)		1.089 (1.131)		0.259 (0.4385)	
	rs7593500	*G*	*0.908*	0.910 (0.9046)	*0.744*	0.745 (0.7404)	*1.108*	1.111 (1.105)	*0.343*	0.353 (0.3262)	*1*
	rs1396919	C	0.892	0.89	0.726	0.72	1.095	1.09	0.273	0.24	1
	rs966859 *	G	0.795	0.799 (0.7939)	0.635	0.638 (0.6331)	0.997	1.002 (0.9956)	0.047	0.052 (0.04572)	1
	rs10864934	A	0.845	0.850 (0.8495)	0.699	0.704 (0.7036)	1.021	1.026 (1.026)	0.081	0.090 (0.08981)	0.99
	rs4848377	T		1.027 (1.018)		0.885 (0.8772)		1.193 (1.182)		0.725 (0.8119)	
	rs10496488	*T*	*1.134*	1.135 (1.135)	*0.966*	0.967 (0.9668)	*1.331*	1.332 (1.332)	*0.125*	0.122 (0.1219)	*1*
	rs2420574	G		1.12		0.95		1.31		0.18	
	rs11123277	T		1.12		0.95		1.32		0.17	
	rs12994269	A		1.109 (1.198)		0.903 (0.9635)		1.363 (1.489)		0.324 (0.1041)	
	rs958396	A	0.940	0.93	0.753	0.75	1.175	1.16	0.588	0.54	1
	rs10167935	T		1.059 (1.059)		0.911 (0.9105)		1.232 (1.232)		0.456 (0.4561)	

DPP10	SNPs	minor allele	OR genotyping	OR imputation HM2 (HM3)	CI_L 95% genotyping	CI_L 95% imputation HM2 (HM3)	CI_U 95% genotyping	CI_U 95% imputation HM2 (HM3)	p-value genotyping	p-value imputation HM2 (HM3)	R2
	rs13035561	T		1.079 (1.046)		0.925 (0.9015)		1.258 (1.215)		0.333 (0.5508)	
	rs7587705	A		1.07		0.91		1.26		0.41	
	rs10201854	T		1.09		0.93		1.28		0.29	
	rs12995783	T		1.26		0.91		1.73		0.16	
	rs4277531	*T*	*1.059*	1.059 (1.059)	*0.910*	0.911 (0.9105)	*1.232*	1.232 (1.232)	*0.459*	0.456 (0.4561)	*1*
	rs10496487	*T*	*1.111*	1.111 (1.111)	*0.943*	0.943 (0.9431)	*1.309*	1.309 (1.309)	*0.208*	0.208 (0.2076)	*1*
	rs10496486	G		0.84		0.66		1.05		0.13	
	rs4459743	C	1.052	1.049 (1.046)	0.906	0.904 (0.9015)	1.222	1.218 (1.215)	0.507	0.527 (0.5508)	1
	rs7579207	*A*	*1.078*	1.074 (1.074)	*0.914*	0.911 (0.9107)	*1.271*	1.266 (1.266)	*0.372*	0.397 (0.3968)	*1*
	rs10496485	T		0.94		0.78		1.14		0.54	
	rs7424115	*C*	*0.974*	0.97	*0.728*	0.73	*1.302*	1.3	*0.858*	0.86	*1*
	rs9677654	C		0.791 (0.8382)		0.410 (0.4394)		1.523 (1.599)		0.482 (0.5923)	
	rs983829	T	0.956	0.94	0.729	0.72	1.255	1.23	0.747	0.65	0.99
	rs958457	G	1.036	1.042 (0.9381)	0.874	0.878 (0.7147)	1.229	1.235 (1.231)	0.684	0.639 (0.645)	1
	rs13416481	G		0.895 (0.8833)		0.715 (0.7054)		1.121 (1.106)		0.335 (0.2794)	
	rs2280193	A		0.793 (0.7599)		0.557 (0.5361)		1.130 (1.077)		0.199 (0.123)	
	rs12469615	G		1.03		0.81		1.31		0.8	
	rs17687793	C		1.097 (1.127)		0.741 (0.7702)		1.624 (1.648)		0.644 (0.5387)	
	rs17044138	C		0.8		0.57		1.13		0.21	
	rs17687884	G		0.96		0.59		1.57		0.88	
	rs2420884	A		1.01		0.76		1.35		0.93	
	rs4131302	G	1.027	1.020 (1.024)	0.877	0.872 (0.8746)	1.202	1.194 (1.198)	0.742	0.802 (0.772)	1
	rs10445863	C	1.037	1.032 (1.036)	0.890	0.887 (0.8892)	1.207	1.202 (1.206)	0.645	0.682 (0.6525)	0.98
	rs10195710	A	1.085	1.088 (1.085)	0.932	0.935 (0.932)	1.262	1.266 (1.262)	0.294	0.276 (0.2941)	1
	rs2176250	*G*	*1.040*	1.045 (1.067)	*0.861*	0.865 (0.8818)	*1.257*	1.263 (1.29)	*0.682*	0.649 (0.5065)	*1*
	rs11693764	T		1.028 (1.019)		0.883 (0.8752)		1.197 (1.186)		0.719 (0.8088)	
	rs11675397	G		1.07		0.91		1.25		0.43	
	rs7558702	*A*	*1.119*	1.108 (1.108)	*0.924*	0.916 (0.9158)	*1.356*	1.340 (1.34)	*0.250*	0.292 (0.2923)	*1*
	rs13005061	G		1.08		0.82		1.41		0.58	
	rs7566462	C		0.93		0.78		1.11		0.44	
	rs2067699	G		0.94		0.71		1.26		0.69	
	rs17044170	G		0.903 (0.9275)		0.736 (0.7534)		1.108 (1.142)		0.328 (0.478)	
	rs11123287	A		0.96		0.83		1.12		0.6	
	rs7593121	*G*	*1.031*	1.032 (1.032)	*0.880*	0.882 (0.8815)	*1.208*	1.209 (1.209)	*0.703*	0.692 (0.6921)	*1*
	rs11896538	G		0.87		0.72		1.07		0.18	
	rs956534	A		1		0.85		1.17		0.98	
	rs9308710	T	1.017	0.932 (0.9315)	0.741	0.683 (0.6834)	1.396	1.270 (1.27)	0.917	0.653 (0.6532)	0.99
	rs11123288	A		0.941 (0.949)		0.790 (0.7967)		1.121 (1.13)		0.497 (0.5572)	
	rs10496483	*G*	*1.155*	1.155 (1.136)	*0.894*	0.895 (0.8804)	*1.492*	1.490 (1.465)	*0.269*	0.268 (0.3277)	*1*
	rs17355679	T		1.03		0.88		1.21		0.72	
	rs4610066	G		1.03		0.84		1.25		0.8	
	rs4849387	*G*	*1.143*	1.146 (1.137)	*0.885*	0.888 (0.8809)	*1.478*	1.479 (1.467)	*0.306*	0.295 (0.3244)	*0.99*
	rs10204084	A		1		0.86		1.17		0.96	
	rs6542253	G		0.83		0.32		2.16		0.7	
	rs11123289	G		0.99		0.85		1.15		0.89	
	rs12991531	T		1.002 (0.9699)		0.751 (0.7288)		1.338 (1.291)		0.987 (0.8338)	
	rs6542254	G	0.931	0.928 (0.9277)	0.756	0.754 (0.7535)	1.147	1.142 (1.142)	0.502	0.480 (0.4797)	1
	rs10206444	G		1.012 (1.097)		0.708 (0.7554)		1.446 (1.594)		0.947 (0.6261)	
	rs13001269	*G*	*1.061*	1.061 (1.061)	*0.808*	0.808 (0.8081)	*1.392*	1.392 (1.392)	*0.671*	0.671 (0.6709)	*1*
	rs4849389	A	1.051	1.03	0.819	0.81	1.348	1.31	0.697	0.84	1
	rs13009552	G		1.03		0.82		1.29		0.82	
	rs10496482	G		0.956 (0.9637)		0.823 (0.8287)		1.111 (1.121)		0.558 (0.6311)	
	rs12991665	*A*	*0.959*	0.979 (0.9785)	*0.633*	0.647 (0.6473)	*1.452*	1.479 (1.479)	*0.842*	0.918 (0.9181)	*0.99*
	rs6759005	C		0.99		0.75		1.31		0.95	
	rs7577833	*G*	*0.973*	0.976 (0.9787)	*0.837*	0.839 (0.8422)	*1.130*	1.134 (1.137)	*0.716*	0.747 (0.7788)	*1*
	rs13386690	G		0.970 (0.9773)		0.822 (0.8274)		1.145 (1.154)		0.719 (0.787)	
	rs13033618	G		1.1		0.77		1.58		0.59	
	rs4849390	*T*	*1.003*	1.001 (0.9917)	*0.834*	0.832 (0.8249)	*1.206*	1.203 (1.192)	*0.973*	0.993 (0.9296)	*1*
	rs12619497	*A*	*1.001*	0.979 (0.9785)	*0.664*	0.647 (0.6473)	*1.511*	1.479 (1.479)	*0.995*	0.918 (0.9181)	*0.99*
	rs11690705	A	1.080	1	0.890	0.83	1.310	1.2	0.437	0.99	0.98
	rs6741354	G		0.31		0.03		3		0.31	
	rs9646900	C		1.18		0.68		2.04		0.56	
	rs6542256	C	0.986	0.977 (0.9774)	0.764	0.758 (0.7583)	1.271	1.260 (1.26)	0.911	0.860 (0.8598)	1
	rs908599	G		0.97		0.82		1.15		0.73	
	rs1356383	T		0.93		0.66		1.32		0.7	
	rs2420815	*C*	*1.009*	0.997 (0.9927)	*0.853*	0.843 (0.8397)	*1.193*	1.178 (1.174)	*0.921*	0.967 (0.9318)	*0.99*
	rs10176449	T		1.01		0.86		1.19		0.91	
	rs17044234	A		0.84		0.56		1.26		0.39	
	rs2420819	*T*	*0.992*	0.990 (0.9902)	*0.843*	0.841 (0.8413)	*1.168*	1.165 (1.165)	*0.925*	0.906 (0.9058)	*1*
	rs17823623 *	C		1.62		1.05		2.52		0.03	
	rs17044254	T		0.94		0.62		1.42		0.76	
	rs2421100	*C*	*0.960*	0.960 (0.9595)	*0.814*	0.814 (0.8143)	*1.131*	1.131 (1.131)	*0.622*	0.622 (0.6216)	*1*
	rs870925	*A*	*0.937*	0.937 (0.9369)	*0.806*	0.806 (0.8057)	*1.089*	1.089 (1.089)	*0.397*	0.397 (0.3972)	*1*
	rs9308712	*G*	*1.016*	1.016 (1.016)	*0.833*	0.833 (0.8328)	*1.240*	1.240 (1.24)	*0.875*	0.875 (0.8752)	*1*
	rs13022199	A		1.007 (0.9992)		0.797 (0.7908)		1.271 (1.262)		0.957 (0.9944)	
	rs2421101	C	0.964	0.962 (0.9615)	0.829	0.827 (0.8268)	1.122	1.118 (1.118)	0.638	0.610 (0.6097)	1
	rs17044296	A		1.18		0.71		1.97		0.52	
	rs1155664	*G*	*0.877*	0.873 (0.8727)	*0.715*	0.711 (0.7112)	*1.076*	1.071 (1.071)	*0.210*	0.192 (0.1919)	*1*
	rs17453235	C		1.130 (1.058)		0.909 (0.8464)		1.404 (1.322)		0.272 (0.6214)	
	rs11683441	*T*	*1.002*	0.993 (0.9925)	*0.857*	0.849 (0.8488)	*1.172*	1.161 (1.161)	*0.979*	0.925 (0.9249)	*1*
	rs7561977	A		0.93		0.69		1.27		0.65	
	rs843399	*T*	*0.950*	0.950 (0.95)	*0.803*	0.803 (0.8025)	*1.125*	1.125 (1.125)	*0.551*	0.551 (0.5511)	*1*
	rs952747	C	0.950	0.950 (0.95)	0.803	0.803 (0.8025)	1.125	1.125 (1.125)	0.551	0.551 (0.5511)	1
	**rs1717054 (rs843385 / r^2=1)**	**T**	**1.066**	**1.066 (1.066)**	**0.821**	**0.821 (0.8205)**	**1.384**	**1.384 (1.384)**	**0.633**	**0.633 (0.6334)**	**1**
	rs2030440	T		0.98		0.84		1.14		0.79	
	rs1517363	G		1.19		0.65		2.19		0.58	
	rs843408	G		0.974 (0.9712)		0.836 (0.834)		1.134 (1.131)		0.735 (0.7072)	
	rs843447	T		0.72		0.47		1.09		0.12	
	rs6732466	T		0.98		0.84		1.15		0.79	
	rs1037205	G	0.979	0.979 (0.9859)	0.835	0.835 (0.8407)	1.148	1.148 (1.156)	0.792	0.792 (0.8616)	1
	rs17362720	A		1.094 (1.076)		0.848 (0.8275)		1.411 (1.4)		0.489 (0.583)	
	rs13033419	A	1.018	1.020 (1.019)	0.849	0.851 (0.8504)	1.220	1.222 (1.222)	0.849	0.833 (0.8355)	1
	rs1868327	C	1.015	1.020 (1.02)	0.846	0.851 (0.8506)	1.218	1.222 (1.222)	0.871	0.833 (0.833)	1
	rs17362881	T		0.92		0.72		1.18		0.52	
	rs17453808	T		1.05		0.4		2.75		0.92	
	rs13429901	*T*	*0.962*	0.962 (0.9619)	*0.765*	0.765 (0.7648)	*1.210*	1.210 (1.21)	*0.740*	0.740 (0.7396)	*1*
	rs7584395	*A*	*0.996*	0.996 (1.003)	*0.846*	0.846 (0.8518)	*1.172*	1.172 (1.18)	*0.960*	0.960 (0.9758)	*1*
	rs1971190	C		1.029 (1.032)		0.860 (0.8636)		1.231 (1.234)		0.755 (0.7269)	
	rs1006342	G	0.968	0.968 (0.9616)	0.770	0.770 (0.7648)	1.217	1.217 (1.209)	0.780	0.780 (0.7372)	1
	rs12471022	T		2.531 (1.752)		0.984 (0.6947)		6.507 (4.419)		0.054 (0.2347)	
	rs17044686	A		0.82		0.55		1.23		0.34	
	**rs1545396 (rs7576583 / r^2=1)**	**T**	**0.972**	**0.986 (0.9864)**	**0.739**	**0.750 (0.7501)**	**1.280**	**1.297 (1.297)**	**0.841**	**0.922 (0.9217)**	**1**
	rs1550985	*T*	*1.046*	1.035 (1.042)	*0.887*	0.878 (0.884)	*1.233*	1.221 (1.228)	*0.594*	0.680 (0.6246)	*1*
	rs6738130	*C*	*1.058*	1.042 (1.045)	*0.889*	0.884 (0.8871)	*1.259*	1.229 (1.232)	*0.524*	0.622 (0.5968)	*0.99*
	rs921211	C	1.019	1.019 (1.019)	0.876	0.875 (0.8752)	1.187	1.187 (1.187)	0.805	0.808 (0.8081)	1
	rs17044762	*G*	*0.826*	0.835 (0.8354)	*0.595*	0.603 (0.6029)	*1.146*	1.158 (1.158)	*0.252*	0.280 (0.2798)	*1*
	rs1448472	T		1.03		0.87		1.21		0.76	
	rs12997234 *	G		0.67		0.47		0.94		0.02	
	rs6542278	C		0.99		0.84		1.15		0.85	
	rs10496499	C	0.996	0.975 (0.9753)	0.794	0.783 (0.7827)	1.248	1.215 (1.215)	0.971	0.824 (0.8237)	1
	rs4516432	C	1.037	1.037 (1.037)	0.865	0.865 (0.8654)	1.242	1.242 (1.242)	0.696	0.696 (0.6958)	1
	rs6542281	T		0.91		0.74		1.13		0.4	
	rs6740354	G		0.973 (0.9734)		0.812 (0.8116)		1.167 (1.167)		0.771 (0.771)	
	rs4538204	T		0.970 (0.9631)		0.802 (0.8001)		1.172 (1.159)		0.751 (0.6915)	
	rs12105131	G		1		0.77		1.29		0.98	
	rs4241136	G		1.01		0.86		1.18		0.91	
	rs4848395	C		0.89		0.73		1.08		0.22	
	rs11676361	T		0.94		0.19		4.65		0.94	
	rs4075701	T	0.872	(0.8961)	0.724	(0.7438)	1.050	(1.08)	0.147	(0.2489)	
	rs4404272	A		1.12		0.34		3.7		0.85	
	rs10864943	C	0.934	0.935 (0.9353)	0.786	0.787 (0.787)	1.110	1.112 (1.112)	0.436	0.448 (0.4479)	1
	rs7575868	A		0.95		0.76		1.2		0.69	
	rs11123318	G		0.9		0.77		1.07		0.23	
	rs6542289	A		0.7		0.16		3.14		0.64	
	rs1112923	T		0.93		0.75		1.15		0.51	
	rs10173996	T	0.899	0.879 (0.8788)	0.696	0.681 (0.6814)	1.161	1.133 (1.133)	0.413	0.319 (0.3193)	1
	rs10496501	*C*	*0.966*	0.968 (0.9478)	*0.764*	0.766 (0.7495)	*1.221*	1.223 (1.198)	*0.771*	0.785 (0.6542)	*1*
	rs2421283	C		0.97		0.77		1.23		0.82	
	rs13019180	C	1.090	1.092 (1.092)	0.921	0.923 (0.9227)	1.291	1.292 (1.292)	0.314	0.306 (0.3063)	1
	rs12621324	A		0.958 (0.9774)		0.826 (0.8425)		1.111 (1.134)		0.570 (0.7627)	
	rs7608947	A		1.007 (1.016)		0.865 (0.8727)		1.173 (1.183)		0.927 (0.8382)	
	rs1900282	*T*	*1.108*	1.1	*0.949*	0.94	*1.292*	1.28	*0.194*	0.24	*1*
	rs2901396	*A*	*1.012*	1.024 (1.027)	*0.872*	0.882 (0.884)	*1.176*	1.189 (1.192)	*0.873*	0.758 (0.7301)	*1*
	rs10496504	*G*	*1.122*	1.130 (1.12)	*0.948*	0.954 (0.9458)	*1.328*	1.339 (1.325)	*0.182*	0.157 (0.1895)	*0.98*
	rs10496505	A		0.92		0.77		1.09		0.31	
	rs2034257	*A*	*1.124*	1.13	*0.947*	0.95	*1.333*	1.34	*0.181*	0.16	*1*
	rs1374268	G		1.04		0.9		1.21		0.58	
	rs7608838	A		1.109 (1.043)		0.935 (0.8983)		1.316 (1.211)		0.234 (0.5804)	
	rs17655300	T		1.089 (1.176)		0.670 (0.786)		1.768 (1.76)		0.731 (0.4301)	
	rs17655602	G		0.84		0.56		1.26		0.4	
	rs11682655	G		0.810 (0.8559)		0.634 (0.6611)		1.034 (1.108)		0.091 (0.2375)	
	rs13033021	G		1.081 (1.09)		0.928 (0.9364)		1.260 (1.27)		0.318 (0.2654)	
	rs1446481	*T*	*0.988*	1.01	*0.842*	0.87	*1.159*	1.17	*0.881*	0.94	*1*
	rs2421343	*A*	*0.860*	0.875 (0.8609)	*0.671*	0.684 (0.6732)	*1.102*	1.119 (1.101)	*0.233*	0.286 (0.2327)	*1*
	rs13034936 *	C		1.326 (1.348)		1.021 (1.009)		1.722 (1.799)		0.035 (0.04299)	
	rs736335	C		1.03		0.87		1.23		0.71	
	**rs272071**	**T**		**1.077 (1.09)**		**0.909 (0.9192)**		**1.276 (1.293)**		**0.394 (0.3214)**	

EDN1	SNPs	minor allele	OR genotyping	OR imputation HM2 (HM3)	CI_L 95% genotyping	CI_L 95% imputation HM2 (HM3)	CI_U 95% genotyping	CI_U 95% imputation HM2 (HM3)	p-value genotyping	p-value imputation HM2 (HM3)	R2
	**rs9369217**	**T**		**(0.8512)**		**(0.6868)**		**(1.055)**		**(0.141)**	
	rs1014505	C		0.9		0.78		1.05		0.17	
	rs13198076	A		1.611 (2.076)		0.938 (0.7174)		2.767 (6.007)		0.084 (0.1778)	
	rs9380978	G		0.967 (0.8672)		0.785 (0.7009)		1.192 (1.073)		0.754 (0.1897)	
	**rs1800541**	**-**	**-**	**-**	**-**	**-**	**-**	**-**	**-**	**-**	
	**rs5370 (rs1476046 / r^2=0.95)**	**T**	**0.901**	**0.894 (0.8983)**	**0.743**	**0.738 (0.7411)**	**1.092**	**1.084 (1.089)**	**0.288**	**0.254 (0.2744)**	**1**
	rs9296343	G		1.326 (0.8983)		0.852 (0.7411)		2.064 (1.089)		0.211 (0.2744)	
	rs10478720	T		1.81		0.79		4.15		0.16	
	**rs5370 (rs1800543 / r^2=0.95)**	**T**	**0.901**	**0.89**	**0.743**	**0.74**	**1.092**	**1.08**	**0.288**	**0.25**	**1**
	**rs5369 ***	**A**		**0.733 (0.7403)**		**0.583 (0.5924)**		**0.921 (0.9252)**		**0.0077 (0.008207)**	
	**rs2071942**	**-**	**-**	**-**	**-**	**-**	**-**	**-**	**-**	**-**	
	rs1626492	A		0.977 (0.9255)		0.835 (0.7795)		1.143 (1.099)		0.773 (0.3766)	
	rs6912834	G		1.050 (1.029)		0.834 (0.7684)		1.322 (1.377)		0.677 (0.8495)	
	rs2071943	A		0.826 (0.8983)		0.673 (0.7411)		1.013 (1.089)		0.066 (0.2744)	
	**rs1629862 ***	**A**		**1.61**		**1.08**		**2.39**		**0.02**	
	**rs1630736**	**T**	**1.081**	**1.09**	**0.930**	**0.94**	**1.255**	**1.26**	**0.310**	**0.24**	**1**
	**rs5370**	**T**	**0.901**	**0.894 (0.8983)**	**0.743**	**0.738 (0.7411)**	**1.092**	**1.084 (1.089)**	**0.288**	**0.254 (0.2744)**	**1**
	rs9296344	C		1.100 (0.9691)		0.812 (0.702)		1.491 (1.338)		0.538 (0.8484)	
	rs4714383	T		0.972 (0.9588)		0.830 (0.8197)		1.138 (1.122)		0.725 (0.5991)	
	rs4714384	C		1.038 (0.9784)		0.887 (0.8378)		1.215 (1.143)		0.643 (0.7832)	
	rs2859338	A	1.145	1.155 (1.144)	0.984	0.992 (0.9832)	1.332	1.343 (1.33)	0.080	0.063 (0.08185)	1
	rs7356986	A	1.029	1.029 (1.029)	0.811	0.811 (0.8109)	1.306	1.306 (1.306)	0.814	0.814 (0.8135)	1
	rs11752276 *	C		0.79		0.64		0.98		0.04	

GPR154	SNPs	minor allele	OR genotyping	OR imputation HM2 (HM3)	CI_L 95% genotyping	CI_L 95% imputation HM2 (HM3)	CI_U 95% genotyping	CI_U 95% imputation HM2 (HM3)	p-value genotyping	p-value imputation HM2 (HM3)	R2
	**rs2609234**	**A**	**0.926**	**0.94**	**0.749**	**0.77**	**1.145**	**1.14**	**0.480**	**0.52**	**0.90**
	**rs1006392**	**C**	**1.068**	**1.02**	**0.910**	**0.87**	**1.252**	**1.18**	**0.422**	**0.85**	**0.87**
	**rs1006393 (rs714588 / r^2=0.86)**	**C**	**1.018**	**1.004 (1.018)**	**0.875**	**0.863 (0.8753)**	**1.184**	**1.168 (1.184)**	**0.816**	**0.959 (0.8164)**	**0.99**
	rs1458550	A		1.062 (1.058)		0.906 (0.9021)		1.246 (1.242)		0.458 (0.4861)	
	rs1023556	T		0.976 (0.9762)		0.814 (0.814)		1.171 (1.171)		0.795 (0.7952)	
	rs2530544	T		0.990 (0.9673)		0.821 (0.7975)		1.194 (1.173)		0.917 (0.7354)	
	rs2530545	T		0.971 (0.9845)		0.795 (0.8028)		1.186 (1.207)		0.772 (0.8808)	
	**rs1379928**	**C**	**0.973**	**0.975 (0.9745)**	**0.813**	**0.815 (0.8146)**	**1.164**	**1.166 (1.166)**	**0.762**	**0.778 (0.7778)**	**1**
	**rs963218**	**A**		**0.92**		**0.79**		**1.08**		**0.31**	
	**rs2609215**	**G**	**0.975**	**0.965 (0.9654)**	**0.742**	**0.734 (0.7342)**	**1.281**	**1.269 (1.269)**	**0.854**	**0.801 (0.8012)**	**0.99**
	rs2125404	G		0.920 (0.9114)		0.698 (0.7147)		1.212 (1.162)		0.552 (0.4548)	
	rs1963499	A		0.906 (0.8779)		0.656 (0.6724)		1.251 (1.146)		0.548 (0.3388)	
	rs1419793	C		0.815 (0.5414)		0.395 (0.2119)		1.684 (1.384)		0.581 (0.2)	
	rs2530547	T		1.03		0.88		1.22		0.69	
	rs887020	A		0.97		0.83		1.13		0.7	
	rs2530548	G		1.056 (1.049)		0.899 (0.8958)		1.240 (1.229)		0.505 (0.5513)	
	rs2530552	G		1.034 (0.9845)		0.879 (0.8375)		1.217 (1.157)		0.686 (0.8498)	
	rs411323	T		0.973 (0.9446)		0.823 (0.7989)		1.149 (1.117)		0.743 (0.5051)	
	rs12673132	A		1.028 (1.092)		0.624 (0.6671)		1.693 (1.786)		0.914 (0.7271)	
	rs2530566	G		0.945 (0.9518)		0.812 (0.8186)		1.101 (1.107)		0.467 (0.5205)	
	rs2022142	T		1.03		0.81		1.3		0.83	
	rs2609224	C		0.938 (0.9419)		0.787 (0.7899)		1.119 (1.123)		0.478 (0.5055)	
	rs2530569	A		0.97		0.73		1.27		0.8	
	rs11762584	A		0.980 (0.9926)		0.824 (0.8344)		1.166 (1.181)		0.823 (0.9333)	
	rs1379923	A		0.970 (1.012)		0.813 (0.8493)		1.157 (1.205)		0.735 (0.8969)	
	rs10255979	C		1.057 (1.07)		0.870 (0.8836)		1.283 (1.296)		0.578 (0.4886)	
	rs2531840	A		1.017 (1.013)		0.869 (0.8659)		1.190 (1.186)		0.838 (0.868)	
	rs6957774	C		0.93		0.72		1.2		0.58	
	rs2021374	T		1.11		0.88		1.39		0.39	
	**rs323917**	**G**	**0.961**	**0.95**	**0.698**	**0.67**	**1.323**	**1.35**	**0.807**	**0.76**	**0.74**
	rs765023	G		1.12		0.96		1.31		0.14	
	rs323918	T		1.144 (1.134)		0.985 (0.976)		1.329 (1.318)		0.079 (0.1002)	
	rs17197425	C		1.26		0.85		1.87		0.25	
	rs10250336	A		1.21		0.85		1.72		0.3	
	rs17197589	C		0.94		0.5		1.79		0.85	
	rs2022143	A		1.06		0.89		1.26		0.52	
	rs6956356	C		0.93		0.71		1.22		0.61	
	**rs11773493 (rs740347 / r^2=1)**	**C**	**1.103**	**1.103 (1.113)**	**0.886**	**0.886 (0.9022)**	**1.372**	**1.372 (1.374)**	**0.381**	**0.381 (0.3168)**	**1**
	rs324382	A		1.17		0.96		1.41		0.11	
	rs324393	T		0.99		0.85		1.16		0.88	
	rs13236864	G		1.36		0.95		1.94		0.1	
	**rs324396**	**T**	**0.915**	**0.934 (0.9274)**	**0.776**	**0.793 (0.7872)**	**1.080**	**1.100 (1.093)**	**0.295**	**0.413 (0.3672)**	**1.00**
	rs10243634	A		0.955 (0.9518)		0.821 (0.8186)		1.110 (1.107)		0.546 (0.5208)	
	rs10486656	T		0.91		0.72		1.15		0.43	
	rs10486657	T		1.05		0.86		1.26		0.65	
	rs324976	A		1.01		0.86		1.19		0.9	
	rs324986	C		0.968 (0.9734)		0.833 (0.8378)		1.125 (1.131)		0.671 (0.7252)	
	rs1585255	G		0.94		0.77		1.14		0.53	
	rs325459	G		1.69		0.57		5.08		0.35	
	rs4580944	T		0.94		0.3		2.92		0.91	
	rs325460	T		1.02		0.88		1.18		0.83	
	rs325468	G		0.964 (0.9813)		0.819 (0.8345)		1.134 (1.154)		0.656 (0.819)	
	rs725902	G		1.01		0.86		1.19		0.9	
	rs17789642	A		0.991 (1.008)		0.772 (0.7843)		1.274 (1.296)		0.946 (0.9486)	
	rs1419868	T		0.956 (0.9488)		0.821 (0.8146)		1.114 (1.105)		0.567 (0.4995)	
	rs17170012	G		0.943 (0.9168)		0.724 (0.6897)		1.228 (1.219)		0.663 (0.5496)	
	rs12701388	A		2.82		0.57		14.04		0.21	
	rs17200135	C		1.068 (0.9344)		0.647 (0.473)		1.764 (1.846)		0.797 (0.8451)	
	rs17170013	G		1.22		0.53		2.8		0.64	
	rs17789834	G		0.911 (0.9687)		0.750 (0.7969)		1.106 (1.178)		0.344 (0.7498)	
	rs1860369	T		1.14		0.94		1.37		0.17	
	rs6947841	T		0.920 (0.9348)		0.758 (0.7773)		1.118 (1.124)		0.402 (0.4739)	
	rs17170017	G		1.05		0.89		1.25		0.57	
	rs10249085	T		0.905 (0.9342)		0.770 (0.7994)		1.064 (1.092)		0.226 (0.3922)	
	rs9648043	A		0.91		0.77		1.08		0.29	
	rs17790181	C		0.989 (0.9695)		0.832 (0.8148)		1.175 (1.154)		0.898 (0.7272)	
	rs10250709	A	0.938	0.936 (0.9334)	0.802	0.801 (0.7984)	1.097	1.095 (1.091)	0.423	0.408 (0.3871)	1
	rs2392285	A		0.89		0.69		1.14		0.36	
	rs10238983	C	0.944	0.958 (0.9577)	0.794	0.805 (0.8051)	1.124	1.139 (1.139)	0.519	0.625 (0.6254)	0.99
	rs2040854	C		1.11		0.92		1.33		0.27	
	rs1833090	A	1.049	1.049 (1.049)	0.882	0.882 (0.882)	1.249	1.249 (1.249)	0.586	0.586 (0.5864)	1
	rs10257456	C		0.89		0.76		1.03		0.12	
	rs10271686	*G*	*0.944*	0.943 (0.943)	*0.806*	0.805 (0.8051)	*1.106*	1.105 (1.105)	*0.477*	0.467 (0.4671)	*1*
	rs11771425	G	0.863	0.851 (0.8584)	0.667	0.659 (0.6642)	1.116	1.100 (1.109)	0.261	0.218 (0.2435)	1
	rs7804942	*G*	*0.940*	0.937 (0.9426)	*0.811*	0.808 (0.8128)	*1.090*	1.087 (1.093)	*0.414*	0.393 (0.4346)	*1*
	rs1859409	*T*	*1.044*	1.047 (1.043)	*0.891*	0.894 (0.8902)	*1.223*	1.227 (1.222)	*0.597*	0.570 (0.6021)	*1*
	**rs323922**	**C**	**1.093**		**0.935**		**1.278**		**0.264**		
	**rs324377**	**A**	**1.128**		**0.965**		**1.317**		**0.130**		
	**rs324384**	**-**	**-**	**-**	**-**	**-**	**-**	**-**	**-**	**-**	
	rs1649220	*A*	*0.935*	0.935 (0.9325)	*0.804*	0.804 (0.8018)	*1.088*	1.088 (1.084)	*0.385*	0.385 (0.3642)	*1*
	rs1637673	*G*	*0.869*	0.872 (0.8719)	*0.741*	0.744 (0.7435)	*1.019*	1.022 (1.022)	*0.084*	0.091 (0.09148)	*1*
	rs328906	*A*	*1.136*	1.136 (1.141)	*0.938*	0.938 (0.942)	*1.376*	1.376 (1.382)	*0.193*	0.193 (0.1775)	*1*
	rs2023328	*A*	*1.081*	1.091 (1.091)	*0.887*	0.895 (0.8953)	*1.318*	1.329 (1.329)	*0.441*	0.389 (0.3886)	*1*
	rs329240	*T*	*0.873*	0.869 (0.8664)	*0.744*	0.741 (0.7389)	*1.024*	1.019 (1.016)	*0.095*	0.084 (0.07752)	*1*
	rs329252	*T*	*0.924*	0.919 (0.9193)	*0.794*	0.791 (0.7905)	*1.075*	1.069 (1.069)	*0.305*	0.275 (0.2745)	*1*
	rs329263	*C*	*0.924*	0.922 (0.922)	*0.794*	0.793 (0.7927)	*1.076*	1.072 (1.072)	*0.309*	0.292 (0.2919)	*1*
	rs329580	*C*	*0.897*	0.897 (0.8969)	*0.775*	0.775 (0.7747)	*1.038*	1.038 (1.038)	*0.145*	0.145 (0.1451)	*1*

GSTP1	SNPs	minor allele	OR genotyping	OR imputation HM2 (HM3)	CI_L 95% genotyping	CI_L 95% imputation HM2 (HM3)	CI_U 95% genotyping	CI_U 95% imputation HM2 (HM3)	p-value genotyping	p-value imputation HM2 (HM3)	R2
	rs7927381	T		1.273 (1.242)		0.981 (0.9596)		1.654 (1.608)		0.070 (0.09953)	
	rs614080 **	A	0.778 /	0.778 (0.7779)	0.668 /	0.668 (0.6679)	0.906 /	0.906 (0.906)	0.0013 /	0.0012 (0.0012)	1
	rs6591255	A	1.159 /	(1.192)	0.990 /	(1.024)	1.356 /	(1.388)	0.066 /	(0.02357)	
	rs6591256 *	G	1.190 /	1.18	1.022 /	1.01	1.387 /	1.37	0.026 /	0.03	0.99
	rs1695	G	1.125 /	1.165 (1.212)	0.962 /	0.996 (1.034)	1.316 /	1.362 (1.421)	0.141 /	0.056 (0.01765)	0.94
	rs1799811	T	0.985 /	1.04	0.758 /	0.81	1.279 /	1.35	0.908 /	0.76	1
	rs1871042	T		1.161 (1.227)		0.993 (1.047)		1.358 (1.439)		0.061 (0.0116)	

IL12B	SNPs	minor allele	OR genotyping	OR imputation HM2 (HM3)	CI_L 95% genotyping	CI_L 95% imputation HM2 (HM3)	CI_U 95% genotyping	CI_U 95% imputation HM2 (HM3)	p-value genotyping	p-value imputation HM2 (HM3)	R2
	rs6870828	C		0.94		0.81		1.09		0.4	
	rs1865014	T		0.92		0.76		1.12		0.43	
	rs3181224	C	1.013 /	1.01	0.797 /	0.8	1.289 /	1.29	0.914 /	0.91	1
	rs3212227	G		1.09		0.9		1.32		0.4	
	rs3213120	T		1.21		0.77		1.9		0.41	
	rs3213102 *	T		3.04		1.11		8.35		0.03	
	rs2853694	T		1.028 (1.028)		0.884 (0.8844)		1.195 (1.195)		0.718 (0.7184)	
	rs3213096	T		5.656 (2.12)		0.679 (0.6498)		47.100 (6.918)		0.109 (0.2129)	
	rs3213094	T		1.08		0.89		1.31		0.43	
	rs2569253	T	1.012 /	0.988 (0.9913)	0.868 /	0.847 (0.8496)	1.181 /	1.153 (1.157)	0.877 /	0.879 (0.9112)	1
	rs2569254	T	0.953 /	0.955 (0.9603)	0.784 /	0.787 (0.7906)	1.157 /	1.160 (1.166)	0.624 /	0.646 (0.6827)	1
	rs2546891	A		3.78		0.8		17.84		0.09	
	rs3181216	T	1.023 /	1.02	0.866 /	0.87	1.209 /	1.2	0.789 /	0.79	0.97
	rs3213117	C		1.05		0.69		1.61		0.82	
	rs1003199	T		1.013 (0.9847)		0.868 (0.8438)		1.181 (1.149)		0.872 (0.8449)	
	rs1433048	G	0.884 /	0.872 (0.8816)	0.724 /	0.713 (0.7221)	1.080 /	1.065 (1.076)	0.227 /	0.180 (0.2159)	0.98
	rs2546893	A		1.01		0.86		1.17		0.94	
	rs730691	T	0.983 /	0.982 (0.9818)	0.837 /	0.836 (0.836)	1.154 /	1.153 (1.153)	0.831 /	0.821 (0.8228)	1
	rs2546890	G	0.980 /	0.966 (0.9685)	0.839 /	0.828 (0.8308)	1.144 /	1.126 (1.129)	0.795 /	0.655 (0.6831)	1
	rs10052709	G		0.98		0.79		1.23		0.89	
	rs1422876	C		0.94		0.8		1.09		0.41	

IL13	SNPs	minor allele	OR genotyping	OR imputation HM2 (HM3)	CI_L 95% genotyping	CI_L 95% imputation HM2 (HM3)	CI_U 95% genotyping	CI_U 95% imputation HM2 (HM3)	p-value genotyping	p-value imputation HM2 (HM3)	R2
	rs3091307	G		1.11		0.93		1.33		0.24	
	rs1881457	C	1.076 /	(1.12)	0.898 /	(0.9393)	1.291 /	(1.334)	0.427 /	(0.2074)	
	rs1800925	T	1.085		0.904		1.303		0.380		
	rs1295686 *	A	1.220 /	1.230 (1.23)	1.016 /	1.024 (1.024)	1.464 /	1.478 (1.478)	0.033 /	0.027 (0.02681)	0.99
	rs20541 *	T	1.227 /	1.225 (1.221)	1.021 /	1.020 (1.016)	1.475 /	1.472 (1.468)	0.029 /	0.030 (0.03319)	1
	rs1295683 *	A		1.26		1		1.59		0.05	
	rs2243297	A		1.39		0.92		2.11		0.12	
	rs2243204	T	1.129 /	1.129 (1.129)	0.870 /	0.870 (0.8701)	1.464 /	1.464 (1.464)	0.362 /	0.362 (0.3616)	1
	rs2243298	A		2.15		0.7		6.56		0.18	

IL4	SNPs	minor allele	OR genotyping	OR imputation HM2 (HM3)	CI_L 95% genotyping	CI_L 95% imputation HM2 (HM3)	CI_U 95% genotyping	CI_U 95% imputation HM2 (HM3)	p-value genotyping	p-value imputation HM2 (HM3)	R2
	rs762534	A		1.14		0.86		1.51		0.37	
	rs2243240	T		1.62		0.63		4.13		0.32	
	rs2243248	G		0.998 (0.9442)		0.772 (0.6999)		1.291 (1.274)		0.990 (0.7071)	
	rs2243250	T	1.225 /	(1.23)	0.997 /	(1.006)	1.505 /	(1.505)	0.054 /	(0.04348)	
	rs2070874	T	1.221 /	1.356 (1.23)	0.997 /	1.098 (1.006)	1.494 /	1.675 (1.505)	0.054 /	0.005 (0.04348)	0.77
	rs2243263	C		0.98		0.76		1.25		0.84	
	rs2243274 *	A		1.24		1.01		1.52		0.04	
	rs2406539	T		1.16		0.98		1.37		0.09	

IL4R	SNPs	minor allele	OR genotyping	OR imputation HM2 (HM3)	CI_L 95% genotyping	CI_L 95% imputation HM2 (HM3)	CI_U 95% genotyping	CI_U 95% imputation HM2 (HM3)	p-value genotyping	p-value imputation HM2 (HM3)	R2
	rs8057585	C		1.104 (1.097)		0.946 (0.9404)		1.288 (1.28)		0.209 (0.2388)	
	rs2057768 *	T	1.267 /	1.119 (1.106)	1.061 /	0.944 (0.9335)	1.512 /	1.326 (1.31)	0.0089 /	0.195 (0.2443)	0.89
	rs6498012	C		1.080 (1.071)		0.922 (0.9145)		1.263 (1.253)		0.340 (0.3959)	
	rs1110470	A		0.89		0.76		1.03		0.12	
	rs4787948	G	1.110 /	1.111 (1.115)	0.940 /	0.940 (0.9434)	1.312 /	1.312 (1.317)	0.218 /	0.217 (0.2022)	1
	rs2283563	A	0.896 /	0.893 (0.8928)	0.761 /	0.759 (0.7588)	1.054 /	1.050 (1.05)	0.185 /	0.172 (0.1719)	1
	rs3024530	G	1.134 /	1.134 (1.134)	0.975 /	0.975 (0.9748)	1.319 /	1.319 (1.319)	0.103 /	0.103 (0.1033)	1
	rs3024536	T	1.091 /	1.091 (1.085)	0.889 /	0.889 (0.8842)	1.338 /	1.338 (1.331)	0.406 /	0.406 (0.4348)	1
	rs1805010 *	G	1.234 /	(1.149)	1.053 /	(0.9865)	1.446 /	(1.337)	0.0093 /	(0.07421)	
	rs3024560	G		1.151 (1.157)		0.985 (0.9903)		1.344 (1.352)		0.078 (0.06614)	
	rs2301807	A		0.85		0.6		1.2		0.36	
	rs2239349	A		0.933 (0.8096)		0.542 (0.5906)		1.609 (1.11)		0.804 (0.1893)	
	rs2239346 *	A		0.84		0.72		0.98		0.02	
	rs3116578	T		1.9		0.85		4.25		0.12	
	rs3024585 *	A	1.180 /	1.179 (1.175)	1.014 /	1.013 (1.01)	1.373 /	1.371 (1.366)	0.032 /	0.033 (0.03679)	1
	rs3024613	T		0.869 (0.8786)		0.747 (0.756)		1.010 (1.021)		0.067 (0.09138)	
	rs3024620	G		0.86		0.6		1.23		0.41	
	rs3024622 *	G		1.22		1.04		1.42		0.01	
	rs3024630	G	0.932 /	0.946 (0.9454)	0.724 /	0.736 (0.7348)	1.199 /	1.215 (1.216)	0.582 /	0.661 (0.6621)	0.99
	rs3024691	A		0.56		0.13		2.35		0.43	
	rs3024647	G		0.94		0.76		1.16		0.55	
	rs3024660	C	0.910 /	0.943 (0.912)	0.741 /	0.765 (0.742)	1.118 /	1.163 (1.121)	0.371 /	0.584 (0.3811)	0.96
	rs2234897	C		0.85		0.52		1.39		0.51	
	rs1805011	C	1.114 /	1.103 (1.103)	0.870 /	0.862 (0.8624)	1.428 /	1.411 (1.411)	0.392 /	0.435 (0.4346)	1
	rs1805011 (rs2234898 / r²=0.95)	C	1.114 /	1.103 (1.104)	0.870 /	0.862 (0.8628)	1.428 /	1.411 (1.413)	0.392 /	0.435 (0.4311)	1
	rs1805012	C	1.111 /	1.104 (1.104)	0.865 /	0.863 (0.8628)	1.427 /	1.413 (1.413)	0.411 /	0.431 (0.4311)	1
	rs2234900	C	- /	(1.104)	- /	(0.8628)	- /	(1.413)	- /	(0.4311)	
	rs1805015	C	1.035 /	1.030 (1.024)	0.842 /	0.838 (0.8293)	1.272 /	1.265 (1.264)	0.744 /	0.782 (0.828)	1
	rs1801275	G	1.052 /	1.05	0.874 /	0.87	1.267 /	1.27	0.589 /	0.59	1
	rs1805016	G		0.848 (0.9305)		0.602 (0.6517)		1.195 (1.328)		0.347 (0.6917)	
	rs2074570	C		1.13		0.77		1.67		0.53	
	rs1049631	G	1.083 /	1.084 (1.084)	0.931 /	0.932 (0.9315)	1.260 /	1.260 (1.26)	0.301 /	0.298 (0.2983)	1
	rs3024698	A		0.85		0.46		1.57		0.6	
	rs3024685	C		1.020 (1.027)		0.874 (0.8805)		1.190 (1.198)		0.802 (0.7353)	
	rs12102586	T		1.04		0.8		1.36		0.78	
	rs4787956	G		1.037 (1.049)		0.886 (0.8969)		1.214 (1.227)		0.650 (0.5495)	

PTGDR	SNPs	minor allele	OR genotyping	OR imputation HM2 (HM3)	CI_L 95% genotyping	CI_L 95% imputation HM2 (HM3)	CI_U 95% genotyping	CI_U 95% imputation HM2 (HM3)	p-value genotyping	p-value imputation HM2 (HM3)	R2
	rs803014	A		1.07		0.86		1.33		0.56	
	rs803012 (rs708487 / r²=1)	C	0.939 /	0.941 (0.9412)	0.784 /	0.786 (0.7863)	1.124 /	1.127 (1.127)	0.493 /	0.509 (0.5087)	1
	rs803011	T	0.945		0.786		1.137		0.552		
	rs8004654	T	0.913	(0.9531)	0.782	(0.8204)	1.066	(1.107)	0.250	(0.5302)	
	rs11157907	C	0.975		0.788		1.207		0.816		
	rs11851957	T	0.957 /	0.957 (0.9516)	0.775 /	0.775 (0.7703)	1.182 /	1.182 (1.176)	0.684 /	0.684 (0.6457)	1
	rs17831669	C		1.134 (1.111)		0.866 (0.8503)		1.484 (1.453)		0.361 (0.4392)	
	rs708486	C	0.987 /	0.99	0.847 /	0.85	1.150 /	1.15	0.866 /	0.87	1
	rs810633 (rs803010 / r²=0.85)	T	1.042 /	1.042 (1.042)	0.879 /	0.879 (0.8785)	1.235 /	1.235 (1.235)	0.639 /	0.639 (0.6391)	1
	rs12895027	T		1.155 (1.581)		0.651 (0.8259)		2.048 (3.025)		0.623 (0.1669)	
	rs1953367	C	1.012 /	1.014 (1.005)	0.848 /	0.849 (0.8428)	1.207 /	1.210 (1.199)	0.898 /	0.881 (0.9552)	0.99

TNF	SNPs	minor allele	OR genotyping	OR imputation HM2 (HM3)	CI_L 95% genotyping	CI_L 95% imputation HM2 (HM3)	CI_U 95% genotyping	CI_U 95% imputation HM2 (HM3)	p-value genotyping	p-value imputation HM2 (HM3)	R2
	rs915654	A		1.12		0.95		1.31		0.18	
	rs2844482 (rs1800630 / r²=0.89)	A	1.145 /	1.170 (1.17)	0.940 /	0.962 (0.9615)	1.396 /	1.423 (1.423)	0.179 /	0.117 (0.1169)	1
	rs2857713	G	1.084 /	1.08	0.917 /	0.92	1.281 /	1.28	0.347 /	0.35	1
	rs3093543	C	1.124		0.835		1.515		0.441		
	rs1041981	A	1.172 /	1.17	0.992 /	0.99	1.384 /	1.38	0.061 /	0.07	1
	rs1799964	C	1.146 /	1.121 (1.126)	0.955 /	0.939 (0.9432)	1.375 /	1.338 (1.345)	0.144 /	0.206 (0.189)	0.92
	rs1799724	T	0.835		0.649		1.075		0.163		
	rs1800750	A		0.623 (1.493)		0.104 (0.7545)		3.740 (2.955)		0.605 (0.2497)	
	rs1800629 *	A	1.333 /	1.337 (1.385)	1.074 /	1.086 (1.122)	1.654 /	1.646 (1.709)	0.0091 /	0.0062 (0.0024)	0.93
	rs361525	-	-	-	-	-	-	-	-	-	
	rs3093662	G	0.977 /	0.977 (0.977)	0.729 /	0.729 (0.7293)	1.307 /	1.307 (1.307)	0.873 /	0.873 (0.873)	1
	rs3093668	C		0.95		0.67		1.35		0.77	
	rs3093672	T		0.466 (0.864)		0.116 (0.4967)		1.870 (1.504)		0.281 (0.606)	
	rs3093561	A		1.88		0.56		6.28		0.3	
	rs769177 *	T		0.583 (0.599)		0.385 (0.3937)		0.883 (0.914)		0.011 (0.017)	
	rs3093559	T		1.096 (1.089)		0.629 (0.6312)		1.911 (1.88)		0.747 (0.758)	

VDR	SNPs	minor allele	OR genotyping	OR imputation HM2 (HM3)	CI_L 95% genotyping	CI_L 95% imputation HM2 (HM3)	CI_U 95% genotyping	CI_U 95% imputation HM2 (HM3)	p-value genotyping	p-value imputation HM2 (HM3)	R2
	rs12721364	A		0.896 (0.9297)		0.723 (0.7472)		1.110 (1.157)		0.313 (0.5132)	
	rs7968585 (rs7975232 / r²=0.86)	T	1.028 /	1.034 (1.034)	0.883 /	0.889 (0.8889)	1.196 /	1.203 (1.203)	0.722 /	0.665 (0.6649)	1
	rs6580639	C		0.7		0.47		1.04		0.08	
	rs12721385	C		1.21		0.6		2.46		0.59	
	rs11574149	T		1.01		0.46		2.24		0.97	
	rs3858733	G		0.756 (0.6768)		0.361 (0.4104)		1.584 (1.116)		0.459 (0.1261)	
	rs731236	G	1.063	(1.084)	0.905	(0.9292)	1.247	(1.266)	0.458	(0.3037)	
	rs11574113 *	G		0.78		0.61		1		0.05	
	rs757343	A	0.797 /	0.797 (0.7973)	0.630 /	0.630 (0.6297)	1.010 /	1.010 (1.01)	0.060 /	0.060 (0.05998)	1
	rs1544410	A	1.089 /	1.093 (1.08)	0.933 /	0.937 (0.9256)	1.270 /	1.276 (1.26)	0.281 /	0.258 (0.3286)	1
	rs7963776	G	1.01 /	0.989 (1.004)	0.850 /	0.847 (0.8612)	1.160 /	1.156 (1.17)	0.890 /	0.891 (0.9629)	
	rs2239185	G	1.012		0.864		1.185		0.886		
	rs11574085 *	A		1.51		1.06		2.15		0.02	
	rs12721367	T		1.19		0.71		2		0.51	
	rs11168267	A		0.76		0.57		1		0.05	
	rs11574077	C		1.137 (1.17)		0.835 (0.849)		1.548 (1.614)		0.416 (0.3367)	
	rs2239182	G	1.158 /	1.158 (1.158)	0.995 /	0.995 (0.9954)	1.348 /	1.348 (1.348)	0.057 /	0.058 (0.05733)	1
	rs2107301 **	T	0.755 /	0.767 (0.7512)	0.639 /	0.649 (0.6352)	0.893 /	0.908 (0.8883)	0.0010 /	0.0020 (0.0008)	0.98
	rs2283342	G		0.825 (0.8518)		0.669 (0.6916)		1.018 (1.049)		0.073 (0.1313)	
	rs2239181	G	0.787 /	0.77	0.620 /	0.61	1.000 /	0.98	0.050 /	0.04	0.99
	rs1540339 **	A	0.776 /	0.776 (0.7762)	0.664 /	0.664 (0.6642)	0.907 /	0.907 (0.907)	0.0014 /	0.0014 (0.00143)	1
	rs2239179	G	1.133 /	1.130 (1.127)	0.972 /	0.970 (0.9669)	1.320 /	1.317 (1.313)	0.111 /	0.117 (0.1263)	1
	rs11574066 *	T		1.86		1.02		3.37		0.04	
	rs886441	C	1.163 /	1.158 (1.162)	0.964 /	0.959 (0.963)	1.402 /	1.398 (1.402)	0.116 /	0.128 (0.1173)	1
	rs2189480 *	A	0.845 /	0.841 (0.8319)	0.723 /	0.720 (0.7114)	0.988 /	0.983 (0.9727)	0.035 /	0.029 (0.02104)	1
	rs3819545 **	C	0.786 /	0.791 (0.7868)	0.673 /	0.677 (0.6736)	0.918 /	0.924 (0.919)	0.0023 /	0.0032 (0.0025)	0.99
	rs3782905 *	C	1.204 /	1.18	1.013 /	1	1.431 /	1.39	0.035 /	0.06	0.68
	rs2239186	C	0.864 /	0.864 (0.8687)	0.719 /	0.719 (0.7228)	1.040 /	1.039 (1.044)	0.122 /	0.121 (0.1333)	1
	rs11574049	G		0.655 (1.568)		0.348 (0.5667)		1.230 (4.339)		0.188 (0.3863)	
	rs2254210	A	1.153 /	1.154 (1.154)	0.985 /	0.986 (0.9862)	1.350 /	1.351 (1.351)	0.076 /	0.074 (0.07404)	1
	rs2238136	A	0.862 /	0.867 (0.8667)	0.731 /	0.735 (0.7348)	1.017 /	1.022 (1.022)	0.079 /	0.090 (0.08951)	1
	rs2853564	C	1.134 /	1.132 (1.132)	0.968 /	0.966 (0.9659)	1.330 /	1.327 (1.327)	0.121 /	0.126 (0.1256)	1
	rs4760648	T	0.928 /	0.931 (0.9251)	0.794 /	0.797 (0.7919)	1.084 /	1.088 (1.081)	0.346 /	0.368 (0.3269)	0.99
	rs2853559	A		1.13		0.96		1.33		0.14	
	rs11168287	G		0.88		0.75		1.02		0.09	
	rs4328262	G		0.898 (0.9291)		0.768 (0.7961)		1.049 (1.084)		0.174 (0.3504)	
	rs4334089	A	0.968 /	0.972 (0.9639)	0.814 /	0.818 (0.8108)	1.151 /	1.155 (1.146)	0.712 /	0.747 (0.6767)	1
	rs4237855	G		0.925 (0.9245)		0.789 (0.7899)		1.083 (1.082)		0.333 (0.3286)	
	rs11574026	A		0.96		0.73		1.25		0.74	
	rs3890733	T	1.118 /	1.117 (1.117)	0.951 /	0.950 (0.9503)	1.315 /	1.313 (1.313)	0.177 /	0.180 (0.1795)	1
	rs12427078	G		0.39		0.14		1.1		0.08	
	rs7136534	T		0.991 (0.9593)		0.832 (0.8053)		1.181 (1.143)		0.919 (0.6419)	
	rs12721409	T		1.25		0.43		3.62		0.68	
	rs12581281	T		1.352 (1.209)		0.766 (0.5963)		2.386 (2.45)		0.298 (0.599)	
	rs12721377	C		0.97		0.58		1.6		0.9	
	rs10783219	T		0.9		0.77		1.05		0.19	
	rs11574024	T		0.63		0.39		1.01		0.05	
	rs7299460	T		1.009 (0.9757)		0.852 (0.8234)		1.194 (1.156)		0.920 (0.7764)	
	rs4516035	C	1.105 /	1.113 (1.113)	0.947 /	0.954 (0.9538)	1.290 /	1.299 (1.299)	0.204 /	0.174 (0.1742)	1
	rs11568820	T		0.959 (0.9594)		0.788 (0.7877)		1.168 (1.168)		0.680 (0.6799)	
