# Supplementary material for: Unifying Candidate Gene and GWAS Approaches in Asthma
Source: PLoS One. 2010 Nov 12;5(11):e13894. doi: 10.1371/journal.pone.0013894 (PMC2980484; doi:10.1371/journal.pone.0013894)
Supplement: Table S3 — Comparison of candidate gene SNP coverage by GWAS genotyping systems. Number of genotyped SNPs per candidate gene locus from 5 kb upstream of 1st exon to 5 kb after last exon are given and in brackets () genotyped or covered SNPs with previously reported asthma association. (0.04 MB DOC) [file pone.0013894.s003.doc]

**Supplementary table S3**

| gene | SNPs with previous asthma association | SNPs on Illumina Sentrix HumanHap300 BeadChip | SNPs on Illumina Sentrix Human610-Quad BeadChip | SNPs on Affymetrix GenomeWide 6.0 |
| --- | --- | --- | --- | --- |
| ADAM33 | 18 | 5 (0) | 7 (1) | 5 (3) |
| CCL5 | 2 | 0 (0) | 1 (0) | 7 (1) |
| CD14 | 2 | 0 (0) | 3 (1) | 5 (0) |
| DPP10 | 5 | 143 (4) | 312 (5) | 407 (5) |
| EDN1 | 9 | 4 (5) | 7 (3) | 14 (7) |
| GPR154 | 12 | 15 (5) | 69 (7) | 76 (5) |
| GSTP1 | 2 | 4 (1) | 6 (1) | 3 (0) |
| IL12B | 5 | 6 (2) | 8 (3) | 14 (3) |
| IL13 | 3 | 3 (1) | 3 (1) | 6 (1) |
| IL4 | 3 | 0 (0) | 3 (3) | 9 (3) |
| IL4R | 8 | 12 (5) | 21 (6) | 33 (2) |
| PTGDR | 6 | 5 (3) | 19 (4) | 10 (2) |
| TNF | 5 | 5 (1) | 20 (1) | 3 (1) |
| VDR | 9 | 19 (6) | 32 (6) | 48 (5) |
|  |  |  |  |  |
|  | 89 | 221 (33) | 511 (42) | 640 (38) |
